# What does the brain of children with developmental dyslexia tell us about reading improvement? ERP evidence from an intervention study

**DOI:** 10.3389/fnhum.2014.00441

**Published:** 2014-06-26

**Authors:** Sandra Hasko, Katarina Groth, Jennifer Bruder, Jürgen Bartling, Gerd Schulte-Körne

**Affiliations:** Department of Child and Adolescent Psychiatry and Psychotherapy, University Hospital MunichMunich, Germany

**Keywords:** developmental dyslexia, intervention, treatment, improvement, non-improvement, electrophysiology, N400, N300

## Abstract

Intervention is key to managing developmental dyslexia (DD), but not all children with DD benefit from treatment. Some children improve (improvers, IMP), whereas others do not improve (non-improvers, NIMP). Neurobiological differences between IMP and NIMP have been suggested, but studies comparing IMP and NIMP in childhood are missing. The present study examined whether ERP patterns change with treatment and differ between IMP and NIMP. We investigated the ERPs of 28 children with DD and 25 control children (CON) while performing a phonological lexical decision (PLD) task before and after a 6-month intervention. After intervention children with DD were divided into IMP (*n* = 11) and NIMP (*n* = 17). In the PLD–task children were visually presented with words, pseudohomophones, pseudowords, and false fonts and had to decide whether the presented stimulus sounded like an existing German word or not. Prior to intervention IMP showed higher N300 amplitudes over fronto-temporal electrodes compared to NIMP and CON and N400 amplitudes were attenuated in both IMP and NIMP compared to CON. After intervention N300 amplitudes of IMP were comparable to those of CON and NIMP. This suggests that the N300, which has been related to phonological access of orthographic stimuli and integration of orthographic and phonological representations, might index a compensatory mechanism or precursor that facilitates reading improvement. The N400, which is thought to reflect grapheme-phoneme conversion or the access to the orthographic lexicon increased in IMP from pre to post and was comparable to CON after intervention. Correlations between N300 amplitudes pre, growth in reading ability and N400 amplitudes post indicated that higher N300 amplitudes might be important for reading improvement and increase in N400 amplitudes. The results suggest that children with DD, showing the same cognitive profile might differ regarding their neuronal profile which could further influence reading improvement.

## Introduction

Developmental dyslexia (DD) is characterized by severe problems in learning to read properly and is often accompanied by a comorbid spelling disorder. These difficulties arise unexpectedly, because affected children and adults possess the intelligence, motivation, and educational opportunities required for language acquisition and they do not suffer from neurological or sensory deficits (DSM-5: APA, 2013). With prevalence rates around 4–9%, DD is one of the most common specific developmental disorders (Shaywitz et al., 1990; Katusic et al., 2001; Esser et al., 2002). DD accompanies the individuals throughout their lifespan and interferes with academic achievement and professional success (Shaywitz et al., 1999; Daniel et al., 2006; Willcutt et al., 2007). In addition around 40% of children with DD suffer from comorbid psychiatric disorders, especially from externalizing disorders, low school-related self-esteem, and depressive symptoms, as a consequence of their failure in acquiring adequate reading and spelling skills (Willcutt and Pennington, 2000; Arnold et al., 2005; Daniel et al., 2006; Goldston et al., 2007; Willcutt et al., 2007; Mugnaini et al., 2009). Therefore, the attainment of sustainable intervention effects in children with DD is crucial.

In contrast, the empirical state of research for evidence-based evaluation of interventions for children with DD is low. Current meta-analyses quantified the effectiveness of treatment approaches on reading and spelling disabilities and reported only marginal to average effect sizes (Ise et al., 2012; Galuschka et al., 2014). Because DD has a neurobiological basis (e.g., Shaywitz et al., 2007; Shaywitz and Shaywitz, 2008; Caylak, 2009; Richlan, 2012; Richlan et al., 2013) it is important to understand how interventions work on the neuronal level. Does intervention normalize neuronal activity of children with DD? Or does intervention lead to an enhancement of compensatory mechanisms? A better understanding of treatment related changes on the neuronal level might help to refine intervention programs in order to make treatment more effective.

In addition, meta-analyses reported high heterogeneity between the effect sizes of different studies for both reading and spelling interventions (National Institute of Child Health and Human Development, 2000; Ise et al., 2012; McArthur et al., 2012; Galuschka et al., 2014). Weak and inconsistent effect sizes might amongst others arise by inclusion of participants who do not improve during intervention (non-improvers; NIMP). This assumption is supported by studies indicating that up to 30% of struggling readers do not benefit from intervention (Shanahan and Barr, 1995; Vaughn et al., 2003). A better understanding of neuronal differences between children who improve during intervention (improvers; IMP) and children who continue to struggle might help to predict treatment response and to further establish intervention programs adapted to the special needs of the latter.

Against this background, the aim of the present study was twofold. On the one hand we were interested in investigating which neurophysiological changes occur during treatment. A further goal was to explore whether there might be any pre-existing neurophysiological differences, between IMP and NIMP.

Over the past decade researchers began to focus on the neuronal processes related to inefficient reading and spelling abilities to understand the efficacy of reading and spelling interventions. Treatment-related functional changes have been observed in the neuronal reading network. Aberrant activation patterns in the subsystems of the neuronal reading network including posterior occipito-temporal and parieto-temporal regions as well as inferior-frontal areas in DD have been established (Shaywitz et al., 2007; Shaywitz and Shaywitz, 2008; Caylak, 2009; Richlan, 2012; Richlan et al., 2013). Compared to typically developing children, children with DD show a hypoactivation in the posterior subsystems of the left hemispheric reading network, which was found to be accompanied by an overactivation in homolog right hemispheric regions during performing language tasks (Simos et al., 2002; Demonet et al., 2004; Kronbichler et al., 2007; Shaywitz and Shaywitz, 2008; Richlan et al., 2009). With respect to the inferior-frontal subsystem results are less homogeneous. Some studies report hypoactivation (Paulesu et al., 1996; Wimmer et al., 2010; for meta-analyses see Richlan et al., 2009, 2011) whereas others observed hyperactivation in subjects with DD (Salmelin et al., 1996; Shaywitz et al., 1998; Brunswick et al., 1999; for review see Pugh et al., 2000; Sandak et al., 2004). Furthermore, disconnectivity between posterior and frontal subsystems (Paulesu et al., 1996) as well as the two posterior subsystems (Shaywitz et al., 2002) of the neuronal reading network has been described. After intervention a normalization of activation in the neuronal reading network has been observed in English speaking children (Simos et al., 2002, 2006, 2007b; Aylward et al., 2003; Temple et al., 2003; Shaywitz et al., 2004; Richards et al., 2007; Meyler et al., 2008) and adults with DD (Eden et al., 2004). Furthermore, it has been described that the connectivity between reading-related areas is normalized after treatment (Richards and Berninger, 2008; Keller and Just, 2009). Treatment-related changes have been also found using electrophysiology. Researchers observed changes in several reading-related event-related potential (ERP) measures (MMN: Kujala et al., 2001; Huotilainen et al., 2011; Lovio et al., 2012; P100: Mayseless, 2011; N170: Jucla et al., 2009; Spironelli et al., 2010; P300: Santos et al., 2007; Jucla et al., 2009) as well as in EEG frequency bands (Penolazzi et al., 2010; Weiss et al., 2010) after intervention.

It has been suggested that different neurobiological processing disorders might cause DD and that these differences in brain development within the group of children with DD might further influence improvement in literacy skills during treatment (Noble and McCandliss, 2005). However, studies examining whether there might be neurophysiological differences prior to receiving intervention between IMP and NIMP are less common. To the best of our knowledge only eight studies differentiated between IMP and NIMP (Simos et al., 2005, 2007a; Odegard et al., 2008; Davis et al., 2011; Farris et al., 2011; Rezaie et al., 2011a,b; Molfese et al., 2013).

Six out of these eight studies focused on neuronal differences between IMP and NIMP after intervention. In most studies this was the consequence of applying a cross-sectional design, which investigated neurophysiological activity only after intervention (Odegard et al., 2008; Davis et al., 2011; Farris et al., 2011; Molfese et al., 2013). These cross-sectional studies reported on normal activation patterns throughout the reading network in IMP after intervention or on brain mechanisms which are known to have a compensatory function (Odegard et al., 2008; Davis et al., 2011; Farris et al., 2011; Molfese et al., 2013). In contrast, NIMP who had persistent deficits in reading performance were marked by aberrant activation patterns throughout the reading network (Odegard et al., 2008; Davis et al., 2011), deficiencies in ERP measures (Molfese et al., 2013) and lower functional connectivity between reading-related brain areas (Farris et al., 2011). Furthermore, two longitudinal studies conducted by Simos et al. (2005, 2007a) reported on similar spatial and temporal brain activation patterns in normal developing children and 6–8-year-old (Simos et al., 2005) and 8–10-year-old (Simos et al., 2007a) IMP after intervention, which was not observed in NIMP. However, Simos et al. (2005, 2007a) did not report on pre-existing differences between IMP and NIMP. Small sample sizes and confounding variables such as wide age range probably mask pre-existing differences, which might be expected if different neurobiological processing disorders underlie DD and influence improvement during intervention (Noble and McCandliss, 2005). In line with this assumption, Rezaie et al. (2011a,b) reported on pre-existing differences between adolescent IMP and NIMP using MEG. In contrast to control children (CON) and IMP, children, who did not improve in reading ability displayed reduced activity in left middle- and superior-temporal gyri, left supramarginal and angular gyrus and ventral occipito-temporal regions as well as in the right parahippocampal gyrus (Rezaie et al., 2011a,b). Furthermore, NIMP displayed reduced activity in the superior- and medial-temporal gyrus of both hemispheres compared to CON (Rezaie et al., 2011b). No differences in these areas were found between CON and IMP. Interestingly, the degree of activation in these regions predicted improvement during intervention, suggesting that pre-existing neuronal activity might influence improvement during treatment.

To summarize, neuronal differences between IMP and NIMP have been reported before (Rezaie et al., 2011a,b) and after intervention (Simos et al., 2007a; Odegard et al., 2008; Davis et al., 2011; Farris et al., 2011; Molfese et al., 2013). Even though these studies provide interesting information about IMP and NIMP their informative value is limited due to methodological difficulties. First the cross-sectional design of most studies (Odegard et al., 2008; Davis et al., 2011; Farris et al., 2011; Rezaie et al., 2011a,b; Molfese et al., 2013) makes clear interpretation of the results difficult. Second the inclusion criterion for DD within most of the studies was not very strict (below the 25th for Rezaie et al., 2011a,b; below the 30th percentile for Simos et al., 2007a) or DD was assessed by non-standardized tests (Davis et al., 2011). This suggests that also normally developing children with somewhat poorer reading skills might have participated in previous studies. Third, differentiation between IMP and NIMP was not strict in most studies using either the median split or performance above and below of arbitrary defined percentile ranges in order to group IMP and NIMP (Simos et al., 2005, 2007a; Davis et al., 2011; Rezaie et al., 2011a,b; Molfese et al., 2013). Moreover small sample sizes, wide age ranges (Simos et al., 2007a; Odegard et al., 2008; Farris et al., 2011), differences in reading ability between IMP and NIMP before intervention (Simos et al., 2007a; Odegard et al., 2008; Davis et al., 2011; Farris et al., 2011), partly rehabilitated NIMP (average skills in phonological awareness but not in word reading) and a big time lag between completion of the intervention and participation in the experiments (Odegard et al., 2008; Farris et al., 2011) are further methodological problems which have to be taken into account. In addition to the best of our knowledge, so far nothing has been reported about pre-existing neurophysiological differences between IMP and NIMP in childhood. However, keeping the high number of children, who don't improve during interventions (Shanahan and Barr, 1995; Vaughn et al., 2003; Groth et al., 2013) and the therapy costs involved (Georgii et al., in review) in mind it is absolutely essential to better understand possible markers of improvement and non-improvement.

In order to investigate electrophysiological differences between IMP and NIMP before and after intervention in the present study we took advantage of the phonological lexical decision (PLD)—task. In this task subjects are presented with real words (W), pseudohomophones (PH), pseudowords (PW), and false fonts (FF) and indicate whether the visually presented stimulus sounds like a real word or not (Kronbichler et al., 2007; van der Mark et al., 2009, 2011; Schurz et al., 2010; Wimmer et al., 2010; Hasko et al., 2013). One major advantage of the PLD—task, is the fact, that it is a continuous reading task, which allows to study both orthographic and phonological processing in one experiment (Hasko et al., 2013). The PLD—task taps orthographic processing on two levels. Firstly, by comparing the letter string material (W; PH; PW) to the visual control stimuli (FF) print sensitivity will be examined. Secondly, the contrast between orthographic familiar (W) and unfamiliar (PH; PW) word material, while controlling for phonology in the case of the contrast between W and PH provides information about the subjects' familiarity with orthographic representations. Furthermore, according to dual route models of reading (e.g., Coltheart et al., 1993, 2001) contrasting of unfamiliar (PH; PW) with familiar (W) word material also taps phonological processing because grapheme-phoneme correspondence (GPC) rules need to be applied in order to sound out the orthographic unfamiliar word material (see Hasko et al., 2013).

Using this task we recently proposed a temporal model of reading processes (Hasko et al., 2013) based on the assumption of dual route models of reading (Coltheart et al., 1993, 2001) in normal developing children and we found processing differences in children with DD. According to dual route models of reading (Coltheart et al., 1993, 2001) reading processes take place in a hierarchical manner. After identification of visual features (contrast, color, spatial frequency) of a letter string the first step of reading processes comprises the identification of letters (Coltheart et al., 1993, 2001). Our results show that the first component which is sensitive to print in contrast to non-orthographic stimuli (FF) is the N170 over occipito-temporal electrodes. At about 220 ms CON's N170 mean peak amplitudes are higher for orthographic material compared to FF indicating that letters are identified in this time window. After the identification of letters phonology of a letter string can be accessed in two different ways depending on the orthographic familiarity of the letter string. Familiar known words are read via the lexical route by accessing the orthographic representations in the orthographic lexicon and directly retrieving the corresponding phonological representations from the phonological lexicon. Whereas unfamiliar word forms, such as pseudohomophones and pseudowords or words for which the reader does not possess an entry in the orthographic lexicon are read by applying GPC rules in order to access the phonological representation (Coltheart et al., 1993, 2001). According to dual route models of reading these processes proceed in a parallel manner (Coltheart et al., 1993, 2001) and they occur at about 400 ms (Hasko et al., 2013). In normal developing children N400 amplitudes over centro-parietal electrodes were comparable high for W, PH, and PW suggesting that children rely on comparable reading processes for all letter strings. Thus, with respect to dual route models of reading the N400 might index the process of GPC or the searching process within in the orthographic lexicon. Access to the phonological lexicon in the PLD—task is indexed between 600 and 900 ms by a late positive complex (LPC) over left centro-parietal electrodes, which was higher for phonological familiar W and PH in contrast to PW in normally developing children. Processing differences dependent on the linguistic material in CON were observed only in the LPC, suggesting that similar reading processes were adopted independent of orthographic familiarity. With respect to children with DD our results indicated deficits on all processing steps. Firstly, a diminished mean area under the curve for the word material—FF contrasts in the time window of the N170 indicated that the degree of print sensitivity was reduced in the brain of children with DD. Secondly, reduced N400 amplitudes in children with DD pointed to less specified orthographic representations or impairments in accessing the orthographic lexicon or applying GPC rules. Lastly, the difference between phonological familiar and phonological unfamiliar word material was not found in children with DD suggesting an impaired access to phonological representations or an underspecification of phonological representations.

With respect to the first research question of the present study, namely which neurophysiological changes occur during treatment in children with DD we hypothesized to find effects on the N400. This was expected because the applied intervention programs worked on either orthographic knowledge or GPC, which is reflected by the N400. As found previously (see Hasko et al., 2013) we hypothesized to find higher N400 mean peak amplitudes before intervention for CON in contrast to IMP and NIMP. After intervention we expected that IMP might show an increase in N400 mean peak amplitudes, with the result that differences in N400 mean peak amplitudes between IMP and CON are diminished. No changes in N400 mean peak amplitudes over time were expected for CON and NIMP.

To answer our second research question whether there might be any neurophysiological differences between IMP and NIMP our analysis strategy was exploratory, as to the best of our knowledge there is no study, which allows deriving specific hypotheses regarding ERPs. However, previous MEG studies give us hints that differences between IMP and NIMP might be expected over temporo-parietal areas before intervention.

## Methods

### Participants

As part of a longitudinal study 29 children without DD and 40 children with DD participated in the present study (for detailed description of recruitment procedure see Hasko et al., 2013). All children were tested regarding their reading and spelling abilities before and after intervention by means of German standardized tests. Common word and pseudoword reading fluency was assessed by using the one-minute-fluent reading-test (German: Ein-Minuten-Leseflüssigkeitstest [SLRT-II]; Moll and Landerl, 2010). In this measure, children are presented with a list of common words and pseudowords and are given one minute to read as many items as possible. Spelling was assessed with a basic vocabulary spelling test for grades 2–3 before intervention (German: Weingartener Grundwortschatz Rechtschreib-Test für zweite und dritte Klassen [WRT2+]; Birkel, 1994) and for grades 3–4 after intervention (German: Weingartener Grundwortschatz Rechtschreib-Test für dritte und vierte Klassen [WRT3+]; Birkel, 2007). In addition, reading comprehension was measured with a reading comprehension test for grades 1–6 (German: Leseverständnistest für Erst- bis Sechstklässler [ELFE 1-6]; Lenhard and Schneider, 2006). Moreover, measures of phonological awareness, rapid automatized naming (RAN) of numbers, letters, colors, and objects and working memory (digit span forwards and backwards from the Wechsler Intelligence Scale for Children IV; German: Hamburg-Wechsler-Intelligenztest für Kinder- IV [HAWIK-IV]; Petermann and Petermann, 2007) were taken.

In order to be included into the study the CON's common word reading fluency and spelling performance had to exceed the 25th percentile for both measures. Before intervention both the reading and the spelling score of children with DD had to diverge from the mean *T*-value for at least 1 SD (cutoff criteria was therefore set to a *T*-value of 40) and 1 SD from the IQ according to the regression criterion (Schulte-Körne et al., 2001). Thus, both a discrepancy of reading and spelling abilities from the class or age level, but also from the level expected on the basis of the child's intelligence was required for diagnosing DD. Children with DD were pseudorandomly assigned to one of two intervention programs. Three CON did not take part in the post treatment measurement and one CON had to be excluded from further analyses due to technical problems during EEG recording, resulting in 25 CON. From the children with DD one child started another intervention before our intervention period began and therefore recalled study participation resulting in a sample size of 39 children with DD. In the present study we were interested in the investigation of reading improvement during intervention. Therefore, children with DD were classified as IMP or NIMP after intervention according to their gain in common word reading fluency measured with the SLRT-II. Children were assigned to the group of IMP if their reading ability increased at least half SD from pre to post. We oriented our classification criteria based on results from current meta-analyses reporting effect sizes of *g* = 0.31 and *g* = 0.33 for reading interventions (Ise et al., 2012; Galuschka et al., 2014). Children whose ability did not change at all over time or did decrease from pre to post were classified as NIMP. According to this classification 12 children were identified as IMP, 21 as NIMP and 6 could not be assigned to one of the groups because their gain in common word reading fluency was between 1 and 4 *T*-values. One child from IMP and a total of 4 children from NIMP were excluded from further analyses due to excessive EEG artifacts, resulting in a sample size of 11 IMP and 17 NIMP.

Before intervention all groups had an average age of about 8 years (see Table 1). Gender was distributed similarly in all groups [χ^2^ = 1.35, *p* = 0.51] and apart from 1 IMP and 4 NIMP all subjects were right-handed [χ^2^ = 6.56, *p* = 0.04; see Table 1]. As can be seen in Table 1 all children had an IQ within the normal range (≥ 85 IQ points; as measured with the Culture Fair Intelligence Test; CFT 1; Cattell et al., 1997), the IQ of CON was significantly higher than the IQ of IMP and NIMP (*p* < 0.05). Attention was assessed with the subscale “Attention Problems” of the Child-Behavior-Checklist (CBCL/1–4; Achenbach, 1991). The CBCL-score of all children was below the cut-off score (CBCL-score < 7 for girls and CBCL-score < 8 for boys, see Table 1). In all reading and spelling tests IMP and NIMP performed significantly worse than CON before and after intervention (*p* < 0.001; see Table 1). Furthermore, CON outperformed IMP and NIMP before and after intervention in phoneme deletion, all subtests of the RAN and working memory (*p* < 0.05). The only difference between IMP and NIMP, was found in reading comprehension where IMP performed significantly better than NIMP pre and post (*p* < 0.05). As expected due to group assignment the common word reading fluency increased significantly over time for IMP (*p* < 0.001) and IMP outperformed NIMP in this measure after intervention (*p* < 0.001). Reading comprehension increased in all groups over time (*p* < 0.001). In addition all children improved their performance from pre to post (*p* < 0.05) in phoneme deletion and segmentation and all subtests of the RAN (apart from IMP in the subtest RAN—objects). In order to control for a confounding influence of IQ, handedness and text comprehension on the ERP results the groups were matched according to these variables resulting in sample sizes of 20, 10, and 16 children for CON, IMP, and NIMP, respectively. The Analyses of Variance (ANOVAs) presented below were also run with matched groups and significant results reported below were also observed within these calculations.

**Table 1:** **Descriptive statistics of CON, IMP, and NIMP**.

	**CON (*n* = 25)**		**IMP (*n* = 11)**		**NIMP (*n* = 17)**	
Age	8.18 (0.32)		8.28 (0.39)		8.27 (0.35)	
Sex (male:female)	13:12		8:3		10:7	
Handedness (right:left)	24:1		10:1		13:4	
IQ^a^	112.04 (10.78)		101.55 (6.33)		104.94 (7.57)	
Attention^b^	2.88 (1.83)		4.82 (2.23)		4.35 (2.06)	
	**Pre**	**Post**	**Pre**	**Post**	**Pre**	**Post**
Word reading (T)^c^	56.36 (6.37)	54.28 (5.65)	31.55 (4.13)	38.27 (3.90)	32.24 (3.68)	29.65 (3.39)
Word reading (RS)^c^	58.28 (13.81)	73.76 (13.79)	19.36 (3.14)	39.00 (5.29)	18.53 (3.54)	27.76 (4.71)
Pseudoword reading (T)^c^	54.68 (7.66)	54.56 (9.99)	36.45 (1.51)	37.36 (5.37)	36.29 (3.90)	34.59 (3.71)
Pseudoword reading (RS)^c^	35.44 (7.34)	42.60 (8.58)	17.91 (2.84)	24.64 (5.54)	18.29 (4.07)	21.88 (3.08)
Reading comprehension (T)^d^	57.58 (8.04)	62.42 (4.75)	37.53 (2.68)	44.90 (3.99)	34.65 (2.41)	37.33 (5.55)
Spelling (T)^e^	52.28 (5.34)	55.48 (10.20)	35.27 (2.97)	33.27 (5.80)	33.94 (3.98)	32.76 (4.60)
Phoneme deletion^f^	21.16 (2.98)	23.32 (2.23)	17.09 (4.76)	19.18 (2.99)	17.65 (5.94)	19.35 (4.81)
Phoneme segmentation^g^	4.56 (2.16)	6.32 (2.10)	5.00 (2.10)	5.36 (1.57)	4.88 (2.62)	5.47 (2.38)
RAN – numbers^h^	100.24 (20.69)	114.15 (20.17)	82.20 (10.58)	89.73 (15.49)	78.94 (14.00)	85.51 (14.14)
RAN – letters^h^	104.72 (18.15)	120.33 (18.28)	53.67 (13.41)	59.74 (13.98)	52.07 (17.33)	63.50 (15.79)
RAN – colors^h^	60.03 (10.55)	65.45 (11.69)	49.06 (7.83)	54.62 (8.95)	47.71 (8.49)	52.63 (10.51)
RAN – objects^h^	51.97 (9.30)	60.34 (11.91)	40.99 (11.22)	41.15 (6.98)	37.93 (6.51)	42.43 (7.06)
Working memory, SS^i^	8.36 (2.53)	9.00 (2.72)	7.09 (1.81)	6.55 (1.64)	7.35 (1.54)	6.59 (2.35)

CON, control group; IMP, improvers; NIMP, non-improvers; n, sample size; T, T-values, T-values have a mean of 50 (SD ± 10); RS, raw scores; SS, standard scores, SS have a mean of 10 (SD ± 3);

a CFT 1;

b CBCL/1–4;

c SLRT-II;

d ELFE 1-6;

e WRT 2+/WRT 3+;

f number of correct items, max. 27;

g number of correct items, max. 10;

h items per minute;

i HAWIK-IV.

Parents and children were informed about the aim, purpose, and procedure of the study and gave their written consent prior to inclusion in the study. Before and after intervention children received a present as acknowledgement for their participation in the testing session. Experimental procedures were approved by the Ethical Committee of the Faculty of Medicine at the University of Munich, Germany.

### Intervention

Children with DD received intervention twice a week for 6 month in an individual setting in our clinic. Intervention started in the beginning of the third grade. All children completed 40 units each lasting 45 min. Both intervention programs (IP1 and IP2) were highly structured thus assuring a consistent proceeding between therapists. Furthermore, to ensure fidelity of treatment, therapists, basically students of linguistics and speech therapy, were extensively trained before and regularly supervised during intervention by psychologists and speech and language therapists. In addition video recordings as well as the observation of single treatment sessions were used to assure treatment fidelity.

As mentioned in the section Participants children with DD were pseudorandomly assigned to the treatment groups. IP1 is based on orthographic knowledge and systematic, rule-based strategies (Schulte-Körne and Mathwig, 2007; Ise and Schulte-Körne, 2010; Schulte-Körne et al., 2012). It focuses on the transfer of correct phoneme discrimination and the according orthographic knowledge (e.g., in German orthography long vowels are often marked by a following silent /h/ or another vowel, whereas short vowels are often marked by two following consonants; therefore perceiving the correct vowel length is important for deducing the right orthographic rule). IP2 belongs to the group of phonics trainings (Dummer-Smoch and Hackethal, 2007). Words are read aloud in syllables and phonemes are used instead of letter pronunciation. It focuses on the acquisition of GPC. For this reason only words with a 1-1 GPC are used (for further information see Groth et al., 2013). Six IMP and 8 NIMP did receive IP1 and 5 IMP and 9 NIMP participated in IP2.

### ERP paradigm and procedure

**Figure 1:**
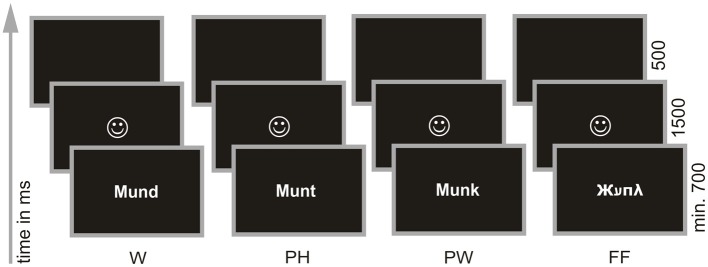
**Phonological lexical decision task**. Words (W; e.g., Mund /mʊnt/, engl.: mouth), pseudohomophones (PH; e.g., Munt /mʊnt/), pseudowords (PW; e.g., Munk /mʊηk/) and false fonts (FF; e.g., Жﬠπ λ) were presented individually in white on black background in the center of a 17 inch screen. Participants were instructed to decide via button press whether a presented stimulus sounded like a real word or not. Figure taken from Hasko et al. (2013).

All children underwent ERP recording before and after intervention (6 month later). During ERP acquisition children performed a PLD—task (Hasko et al., 2013). In this task participants had to decide whether a visually presented stimulus sounded like a real word or not (“Does … sound like a real word?” see Figure 1). Children were presented either with W (orthographically and phonologically familiar forms of German nouns), PH (phonologically correct but orthographically unfamiliar forms of the same words) or PW (phonologically and orthographically unfamiliar forms). W and PH required a “yes” response and PW should be responded with “no.” For each item type (W; PH; PW) 60 stimuli were presented and every item was presented once only. To avoid a response bias toward “yes” responses we included a fourth condition, consisting of 60 FF and requiring a “no” response. FF were created by assigning a FF to each upper and lower case letter. To avoid effects due to item length and complexity all stimuli were matched for number of characters (3–7 characters). In addition W, PH, and PW were controlled for bigram frequency (see Hasko et al., 2013, for a complete list of all stimuli used in the PLD task and for further description of item selection).

All stimuli were presented in white font on black background in the center of a 17″ screen using E-Prime® 2.0 software (Psychology Software Tools, Inc.). The computer screen was placed 70 cm in front of the children resulting in a vertical visual angle of 1.23° and in an average horizontal angle of 3.44°. The 240 stimuli were presented pseudorandomized in four blocks. After each block there was a short break. To ensure that the subjects fully understood the task, the experiment was preceded by a short practice-block (24 trials). Trials utilized in the practice-block did not occur in the experiment. The task was self-paced in order to make sure that even the poorest reader had enough time to read the letter string stimuli. However, all children were presented with the stimuli for a minimum of 700 ms to guarantee that all participants saw the same in the first milliseconds, which is important for ERP analysis. Participants had to decide by button press whether the presented stimulus sounded like a real word or not. Half of the children used their right hand for giving a “yes” response and the left hand for giving a “no” response, the other half used the left hand for “yes” and the right hand for “no” responses. Depending on correct or incorrect response children were provided with a feedback in form of a happy or sad face (1500 ms). The next trial appeared automatically after a blank screen of 500 ms (see Figure 1).

### ERP recording and analysis

**Figure 2:**
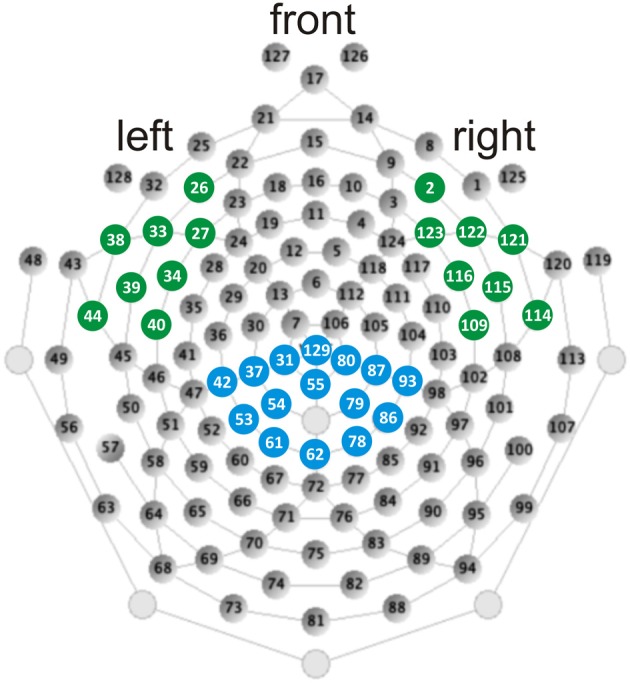
**Illustration of the 128-channel-system and electrode position taken from Electrical Geodesics Inc. (2007)**. Filled blue circles depict electrodes included in the ROI of the N400. Filled green circles depict electrodes included in the LH and RH ROIs of the N300.

EEG was recorded during the stimulus presentation with an Electrical Geodesic Inc. 128-channel-system (see Figure 2, for a schematic illustration of the electrode net). The impedance was kept below 50 kΩ. EEG-data was recorded continuously with Cz as the reference electrode and sampled at 500 Hz. Further analysis steps were performed with Brainvision Analyzer (Brain Products GmbH).

After filtering (low cutoff: 0.5 Hz, time constant 0.3, 12 dB/octave; high cutoff: 40 Hz, 24 dB/octave; Notch filter: 50 Hz; filtered continuous on raw data to avoid discontinuities and transient phenomena), removing EOG-artifacts with Independent Component Analysis (Zhou et al., 2005; Hoffmann and Falkenstein, 2008) and exclusion of other artifacts (gradient criteria: more than 50 μV difference between two successive data points or more than 150 μV in a 200 ms window; absolute amplitude criterion: more than ±150 μV; low activity: less than 0.5 μV in a 100 ms time window), the EEG was re-referenced to the average reference.

The data was then segmented into 1100 ms epochs including 100 ms pre-stimulus baseline and the ERP data was baseline corrected. For inclusion in the statistical analysis a minimum of 20 artifact free trials was necessary. Only correct trials were analyzed. Grand averages of all conditions were computed by averaging separately for each subject group (CON; IMP; NIMP) and each point in time (pre; post).

Based on our hypothesis we were interested in changes of the N400, which reflects GPC or the searching process in the orthographic lexicon. Based on the electrophysiological activity for W in CON before intervention the time window for the N400 was set 330–460 ms using running *t*-tests against zero (*p* < 0.05) at each electrode and the following centro-parietal electrodes were selected for the region of interest (ROI): 31, 37, 42, 53, 54, 55, 61, 62, 78, 79, 80, 86, 87, 93, 129 (see Figure 2, e.g., Deacon et al., 2004; Hasko et al., 2013; for review see Lau et al., 2008; Kutas and Federmeier, 2011).

The analyses run to answer our second research question (whether we could identify any pre-existing electrophysiological differences between IMP and NIMP) was exploratory. During the visual inspection of electrodes and unpaired *t*-tests comparing the electrophysiological activity of IMP and NIMP we observed a hyperactivation over left and right hemispheric (LH and RH) fronto-temporal electrodes starting around 300 ms (see Figure 4). According to the timing and the topography we identified a N300 in the time window of 300–400 ms. Based on the electrophysiological activity for W in CON before intervention using running *t*-tests against zero (*p* < 0.05) at each electrode we selected LH and RH ROIs. Electrodes included in the LH were 26, 27, 33, 34, 38, 39, 40, 44 and electrodes included in the RH were 2, 109, 114, 115, 116, 121, 122, 123 (see Figure 2).

Mean peak amplitude measures capturing data 20 ms before and 20 ms after the individual peak and peak latencies were exported for each electrode of the N400 and N300 ROIs using the defined time windows. The values of individual mean peak amplitudes and peak latencies were averaged after peak export for every ROI.

### Statistical analysis

To test for significant changes over time regarding the N400 mean peak amplitudes and peak latencies we computed ANOVAs. The ANOVAs included the within-subject factors *condition* (W; PH; PW) and *time* (pre; post) and the between-subject factor *group* (CON; IMP; NIMP). For clean ERP data at least 10–20 participants are recommended (Luck, 2005), therefore a further specification of the groups by IP1 and IP2 was not reasonable. In order to test the main hypotheses, namely changes of the N400 during treatment dependent and independent *t*-tests were calculated. Firstly, we hypothesized that CON show higher mean peak amplitudes compared to IMP and NIMP before intervention. Therefore, independent *t*-tests were tested one-sided. Furthermore, we hypothesized that N400 mean peak amplitudes should increase over time in IMP and should remain stable in CON and NIMP, which was also evaluated using one-sided alpha-level.

The expected effect that N400 mean peak amplitudes should increase over time for IMP was moderate to large but only marginally significant. The small sample size (*n* = 11) might be the main reason why the effect did not reach significance on the 5% level. Therefore, we decided to simulate the data for a larger group of IMP. The simulation was done in two steps. Firstly, we estimated the required sample size with g*power using the observed effect size of *d* = 0.54, alpha of 0.05 and beta of 0.95. This estimation resulted in a sample size of 39 IMP. Secondly, the data of 39 IMP was generated with R using normal distribution sampling with the mean and SD of the original IMP group. For each simulated child, 1000 observations were randomly generated and the mean of these observations was calculated.

Similar ANOVAs for repeated measures were computed to analyze the N300 mean peak amplitudes and peak latencies including the additional within-subject factor *hemisphere* (LH; RH). The resulting fourfold interaction between group^*^time^*^condition^*^hemisphere for the N300 mean peak amplitudes was analyzed by stratifying the data on time as we were interested in exploring pre-existing differences between IMP and NIMP. Therefore, two further ANOVAs for repeated measures were calculated separately for pre and post measures. Resulting threefold interactions were analyzed by combining two of the three factors in further ANOVAs for repeated measures. To interpret twofold interactions we ran *post-hoc t*-tests for independent and dependent samples.

The behavioral data (reaction times and accuracy on the PLD—task) was analyzed using ANOVAs for repeated measures including the within-subject factors *condition* (W; PH; PW; FF) and *time* (pre; post) and the between-subject factor *group* (CON; IMP; NIMP). Trials were excluded from analysis if the response times were lower than 200 ms and deviating more than 2.5 SD from the individual group mean within a condition type. This procedure resulted in a loss of 2.65 and 2.96% of the trials for pre and post, respectively. Furthermore, for the reaction time analysis only correct trials were included. Resulting threefold interactions were analyzed by combining two of the three factors in further ANOVAs for repeated measures. To interpret twofold interactions we ran *post-hoc t*-tests for independent and dependent samples.

If sample sizes are equal, ANOVAs are unsusceptible against violations of homogeneity of variance. Given that the sample of CON was bigger than the sample of IMP and NIMP the *F*_max_—test was applied in case of violations of the homogeneity of variances (Bühner and Ziegler, 2009). According to the *F*_max_—test an adjustment of the alpha-level is necessary if the critical value of *F*_max_ > 10 is exceeded (Bühner and Ziegler, 2009). In none of the variables the critical value was exceeded. If necessary the Greenhouse-Geisser correction was applied to correct for violations of the sphericity assumption. The alpha level for all analyses was 0.05. In order to avoid alpha-error-inflation due to multiple comparisons the alpha level of 0.05 for follow-up tests was corrected using the Bonferroni-Holm correction (Bühner and Ziegler, 2009). Bonferroni-Holm correction was applied separately for each set of dependent and independent *t*-tests and for each follow-up ANOVA.

In addition to the *p*-values, effect sizes η^2^_*p*_ for ANOVAs with repeated measures and Cohen's *d* for independent and dependent *t*-tests are reported for significant results (Cohen, 1988; Bühner and Ziegler, 2009). Regarding the ERP data for follow-up tests detailed statistical values will be presented only for significant results, whereas non-significant results are indicated by *p* > 0.05. For the behavioral data significant and non-significant results of the follow-up analyses will be indicated by *p* < 0.05 and *p* > 0.05 without reporting detailed statistical values.

Additionally, in order to better understand the significance of the N300 for improvement during treatment we computed correlations across the whole group of children with DD and for IMP and NIMP separately. Correlations were calculated between N300 mean peak amplitudes before intervention and the gain in common word reading fluency and the N400 after intervention. For common word reading fluency we used the post minus pre differences' of raw scores (see Table 1). Raw scores were used in order to enhance variance. As we did not observe differences between W, PH, and PW in the N400 we decided to use mean values calculated across the three letter string types for the correlation analysis. Because of the small sample size in the IMP group Cook's *d* was calculated for significant correlations in order to check for undue influence of single cases. All cases had a Cook's *d* < 1 indicating that none of the participants had an excessive influence on the correlational results. The correlational analysis was exploratory, therefore Bonferroni-Holm correction was not applied. Significant results on the 5% and tendencies toward significance (10% alpha level) will be reported.

## Results

### N400

#### Mean peak amplitudes

The analysis of the N400 mean peak amplitudes revealed only a main effect group. No main effect time, condition and no interactions could be observed (see Table 2, first column). As no effect of condition could be observed independent and dependent *t*-tests to test our N400 hypotheses were computed across conditions (see Table 3, for N400 mean peak amplitudes).

**Table 2:** **Results of the ANOVAs for repeated measures with *F*-values (df), *p*-values, and effect sizes η^2^_*p*_ for the N400 mean peak amplitudes and latencies including the between-subject factor *group* (CON; IMP; NIMP) and the within-subject-factor *time* (pre; post) and *condition* (W; PH; PW)**.

**Effect**	**Mean peak amplitudes**			**Peak latencies**		
	***F***	***p***	**η^2^_***p***_**	***F***	***p***	**η^2^_***p***_**
Group (G)	5.39 (2, 50)	**0.008**	0.18	4.95 (2, 50)	**0.011**	0.17
Time (T)	0.68 (1, 50)	0.413	–	1.27 (1, 50)	0.265	–
Condition (C)	2.60 (2, 100)	0.080	–	0.49 (2, 100)	0.612	–
G*T	2.59 (2, 50)	0.085	–	2.26 (2, 50)	0.115	–
G*C	0.44 (4, 100)	0.783	–	1.73 (2, 100)	0.150	–
T*C	0.96 (2, 100)	0.388	–	0.50 (2, 100)	0.608	–
G*T*C	1.02 (4, 100)	0.402	–	1.35 (4, 100)	0.258	–

CON, control children; IMP, improvers; NIMP, non-improvers; pre, before intervention; post, after intervention; W, words; PH, pseudohomophones; PW, pseudowords. Significant results are indicated in bold.

**Table 3:**
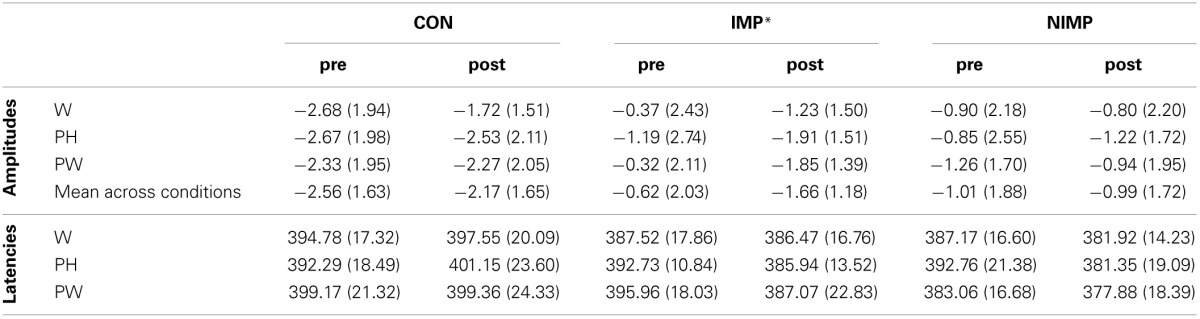
**N400 mean peak amplitudes in μV (SD) and latencies in ms (SD)**.

W, words; PH, pseudohomophones; PW, pseudowords; CON, control children; IMP, improvers; NIMP, non-improvers; pre, before intervention; post, after intervention. ^*^IMP had significantly shorter peak latencies for all conditions before and after intervention (M = 397.38, SD = 16.40) compared to CON (M = 384.02, SD = 10.39).

**Figure 3:**
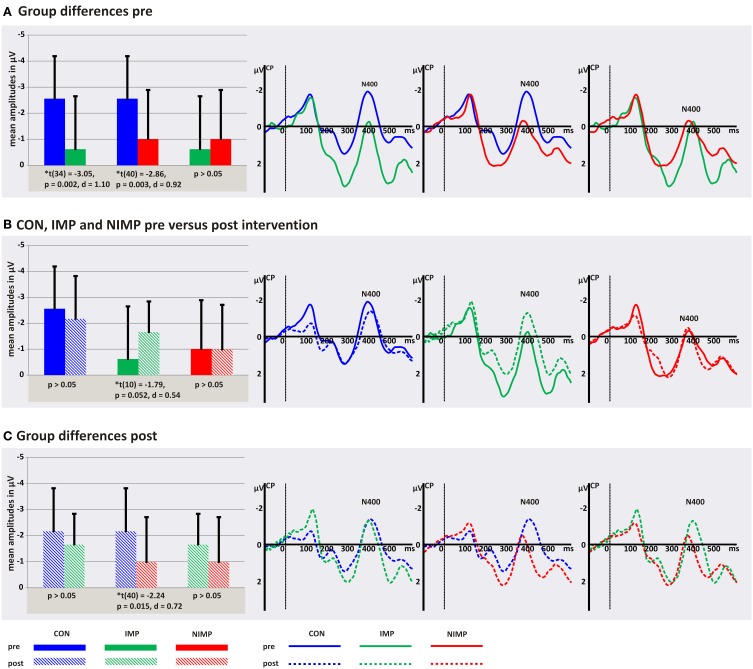
**N400 mean peak amplitudes for control children (CON), improvers (IMP), and non-improvers (NIMP). (A)** Illustrates group differences before intervention (pre). **(B)** Depicts treatment effects. **(C)** Shows group differences after intervention (post). CP = centro-parietal electrodes included in the ROI of the N400. Negativity is depicted upwards. Error bars illustrate standard deviation. ^*^one-sided alpha-level.

In line with our hypothesis independent *t*-tests revealed higher N400 amplitudes for CON compared to IMP and for CON in contrast to NIMP before intervention (see Figure 3A). No difference was found between IMP and NIMP before intervention (see Figure 3A).

Consistent with our expectation a clear trend towards increased N400 mean peak amplitudes in IMP after 6 month of intervention could be observed (see Figure 3B). In agreement with our assumptions N400 mean peak amplitudes remained stable over time in CON and NIMP (see Figure 3B). Mean peak amplitudes were comparable between CON and IMP after intervention but still diminished for NIMP in contrast to CON (see Figure 3C). Even though Table 3 and Figure 3C suggest higher N400 amplitudes in IMP in comparison to NIMP after intervention this effect does not reach significance (see Figure 3C).

***Simulation of the intervention effect in IMP***. Although the increase of the N400 amplitude from pre to post in IMP was moderate to large (*d* = 0.54), this effect was only marginally significant (*p* = 0.052, see Figure 3B). The small sample size (*n* = 11) is probably the main reason why the effect did not reach significance on the 5% alpha level. Therefore, data was simulated for a larger sample size (*n* = 39). Dependent *t*-tests of the simulated data revealed a significant increase in N400 mean peak amplitudes from pre (−0.30 μV ±1.36 SD) to post (−1.81 μV ±0.77 SD), *t*_(38)_ = 6.99, *p* < 0.001, *d* = 1.12.

#### Peak latencies

The analysis of the N400 peak latencies revealed a main effect group (see Table 2, second column). No further effects were observed. Independent *post-hoc t*-tests showed shorter peak latencies for NIMP compared to CON, *t*_(40)_ = 2.97, *p* = 0.005, *d* = 0.96, before and after intervention and no differences in peak latencies were observed between CON and IMP as well as between IMP and NIMP before and after intervention (*p* > 0.05; see Table 3).

### N300

#### Mean peak amplitudes

The analysis of the N300 mean peak amplitudes revealed a main effect group, time, and condition, as well as an interaction condition^*^hemisphere. Furthermore, the four-way interaction group^*^time^*^condition^*^hemisphere reached significance (see Table 4, first column).

**Table 4:** **Results of the ANOVAs for repeated measures with *F*-values, *p*-values, and effect sizes η^2^_*p*_ for the N300 mean peak amplitudes and latencies including the between-subject factor *group* (CON; IMP; NIMP) and the within-subject-factor *time* (pre; post), *condition* (W; PH; PW), and *hemisphere* (LH; RH)**.

**Effect**	**Mean peak amplitudes**			**Peak latencies**		
	**F**	**p**	**η^2^_***p***_**	**F**	**p**	**η^2^_***p***_**
Group (G)	4.76 (2, 50)	**0.013**	0.16	3.11 (2, 50)	0.054	–
Time (T)	4.15 (1, 50)	**0.047**	0.08	0.10 (1, 50)	0.748	–
Condition (C)	4.74 (2, 100)	**0.011**	0.09	0.32 (1.75, 87.58)	0.322	–
Hemisphere (H)	2.11 (1, 50)	0.152	–	0.01 (1, 50)	0.936	–
G*T	1.90 (2, 50)	0.161	–	0.59 (2, 50)	0.556	–
G*C	1.19 (4, 100)	0.319	–	0.76 (3.5, 87.58)	0.537	–
G*H	1.05 (2, 50)	0.358	–	0.08 (2, 50)	0.920	–
T*C	0.35 (2, 100)	0.158	–	0.11 (2, 100)	0.897	–
T*H	3.11 (1, 50)	0.084	–	0.42 (1, 50)	0.521	–
C*H	3.11 (2, 100)	**0.049**	0.06	4.31 (1.78, 89.35)	**0.020**	0.08
G*T*C	0.71 (4, 100)	0.589	–	1.41 (4, 100)	0.236	–
G*T*H	0.13 (2, 50)	0.883	–	0.20 (2, 50)	0.820	–
G*C*H	1.81 (4, 100)	0.132	–	3.01 (3.57, 89.35)	**0.027**	0.11
T*C*H	0.95 (2, 100)	0.389	–	0.79 (2, 100)	0.459	–
G*T*C*H	3.70 (4, 100)	**0.008**	0.13	2.32 (4, 100)	0.062	–

CON, control children; IMP, improvers; NIMP, non-improvers; pre, before intervention; post, after intervention; W, words; PH, pseudohomophones; PW, pseudowords; LH, left hemisphere; RH, right hemisphere. Significant results are indicated in bold.

In order to explore this four-way interaction two separate ANOVAs were conducted for each point in time. The analysis of the N300 mean peak amplitudes before intervention revealed a significant interaction group^*^condition^*^hemisphere, *F*_(4, 100)_ = 3.84, *p* = 0.006, η^2^_*p*_ = 0.13. No main effects and no further interactions could be observed (*p* > 0.05). In order to interpret this three-way interaction separate follow-up ANOVAs were run by combining two of the three factors.

**Figure 4:**
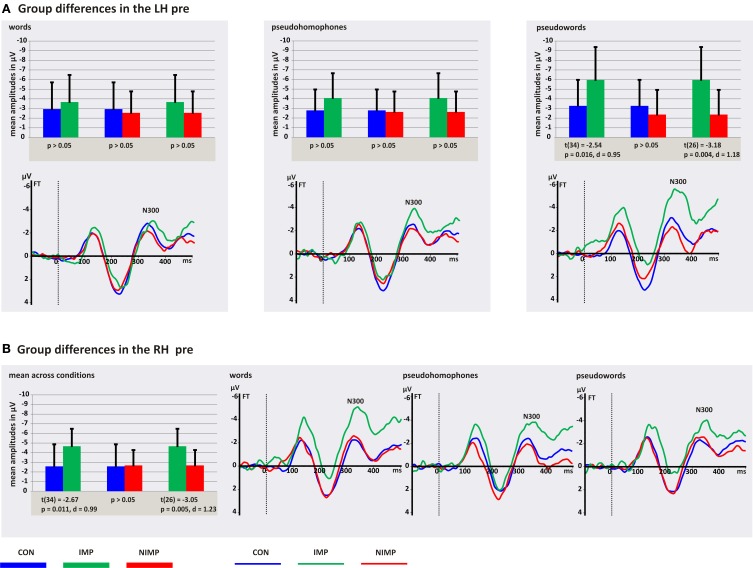
**N300 mean peak amplitudes for control children (CON), improvers (IMP), and non-improvers (NIMP) before intervention. (A)** Illustrates group differences in the left hemisphere (LH). **(B)** Depicts group differences in the right hemisphere (RH). FT = fronto-temporal electrodes included in the LH and RH ROI of the N300. Negativity is depicted upwards. Error bars illustrate standard deviation.

***Follow-up ANOVAs for each hemisphere***. For the LH we found a main effect condition, *F*_(1, 50)_ = 3.84, *p* = 0.015, = 0.08, and an interaction group^*^condition, *F*_(2, 50)_ = 3.05, *p* = 0.020, η^2^_*p*_ = 0.11. No main effect group could be observed (*p* > 0.05). Independent *post-hoc t*-tests revealed that IMP had higher amplitudes for PW in contrast to CON and NIMP in the LH (see Figure 4A). In CON and NIMP amplitudes for PW were comparable high (see Figure 4A). No group differences were found for W and PH (see Figure 4A). Mean amplitudes for W, PH, and PW did not differ within CON, IMP, and NIMP (*p* > 0.05).

For the RH the main effect group, *F*_(2, 50)_ = 4.59, *p* = 0.015, η^2^_*p*_ = 0.16, was significant. No main effect condition and interaction group^*^condition could be observed (*p* > 0.05). Independent *post-hoc t*-tests calculated across conditions revealed higher mean peak amplitudes for IMP in contrast to CON and NIMP (see Figure 4B). No difference was found between CON and NIMP (*p* > 0.05, see Figure 4B).

***Follow-up ANOVAs for each condition***. As could be expected from the ANOVAs run separately for each hemisphere (see above) the analysis revealed a main effect group for PW, *F*_(2, 50)_ = 5.99, *p* = 0.005, η^2^_*p*_ = 0.19. No hemisphere effect as well as no interaction group^*^hemisphere could be observed (*p* > 0.05). Independent *post-hoc t*-tests revealed higher N300 mean peak amplitudes for IMP in contrast to CON, *t*_(34)_ = 2.97, *p* = 0.005, *d* = 1.11 and NIMP, *t*_(26)_ = −3.29, *p* = 0.003, *d* = 1.32, bilaterally and no difference was found between CON and NIMP (*p* > 0.05, see Figures 4A,B). For W and PH no main effects and no interactions were found (*p* > 0.05).

***Follow-up ANOVAs for each group***. A twofold interaction condition^*^hemisphere did occur within the IMP group, *F*_(2, 20)_ = 5.10, *p* = 0.016, η^2^_*p*_ = 0.34, and no main effect condition or hemisphere was observed for the IMP group (*p* > 0.05). This interaction suggests that mean peak amplitudes are higher for PW in contrast to W and PH specifically in the LH (see Figure 4A). However, dependent *post-hoc t*-tests did not reveal amplitude differences between conditions in the LH and RH (*p* > 0.05). Furthermore, mean peak amplitudes were comparable high between the LH and RH for W, PH, and PW (*p* > 0.05). For CON and NIMP no main effects and no interactions were found (*p* > 0.05).

To summarize IMP in contrast to CON and NIMP are marked by higher N300 mean peak amplitudes for all conditions in the RH and additionally for PW in the LH.

**Figure 5:**
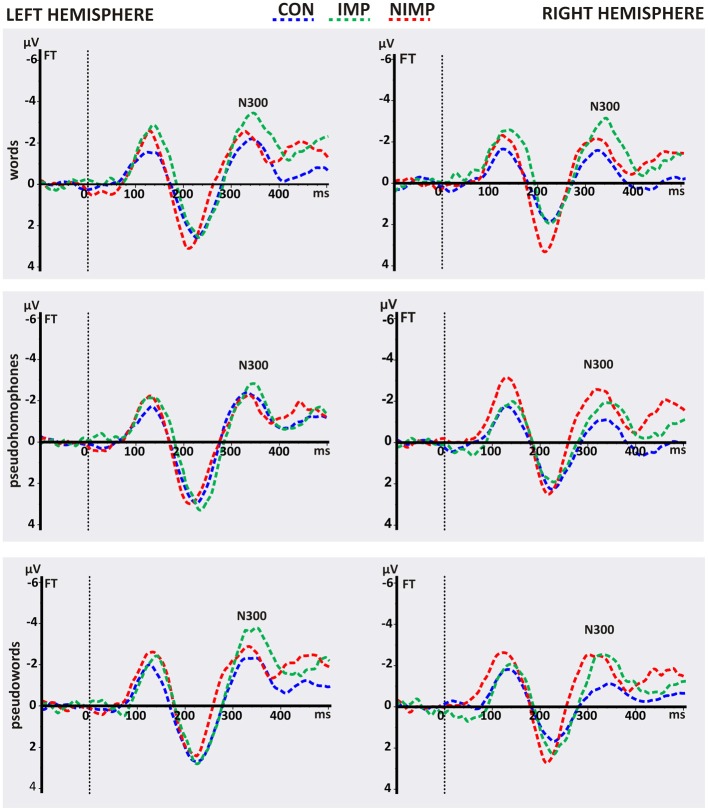
**Illustration of the N300 after intervention**. FT = fronto-temporal electrodes included in the left hemispheric and right hemispheric ROI of the N300 for control children (CON), improvers (IMP), and non-improvers (NIMP). Negativity is depicted upwards.

After intervention no significant main effect group, time, condition and no significant interactions between these factors could be observed for the N300 mean peak amplitudes (*p* > 0.05, see Table 5 and Figure 5).

**Table 5:**
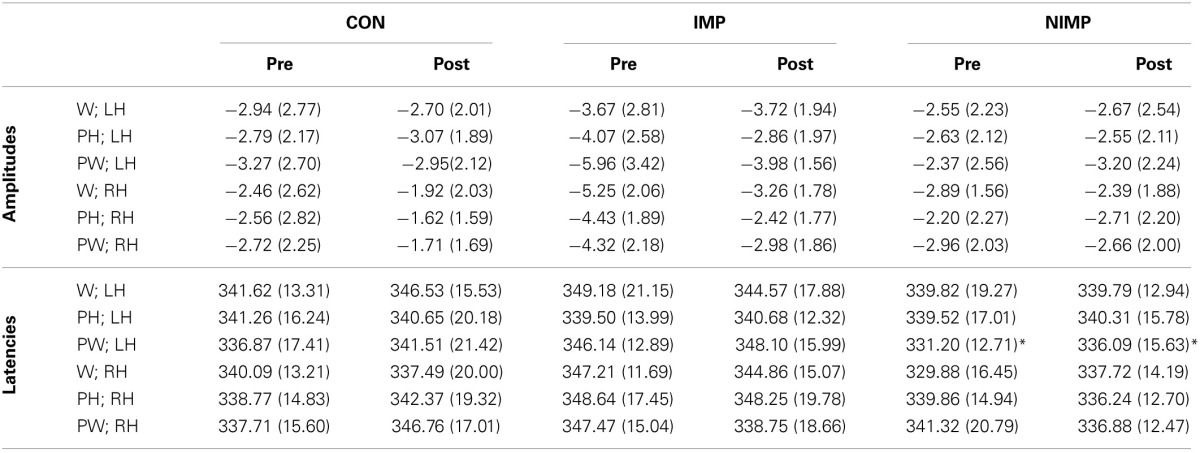
**N300 mean peak amplitudes in μV (SD) and latencies in ms (SD)**.

W, words; PH, pseudohomophones; PW, pseudowords; LH, left hemisphere; RH, right hemisphere; CON, control children; IMP, improvers; NIMP, non-improvers; pre, before intervention; post, after intervention. ^*^In NIMP PW in the LH over pre and post (M = 333.64, SD = 11.31) are significantly smaller compared to W (M = 339.81, SD = 11.50) and PH (M = 339.91, SD = 11.28).

#### Peak latencies

The analysis of the N300 peak latencies revealed a twofold interaction condition^*^hemisphere and a threefold interaction group^*^condition^*^hemisphere (see Table 4, second column). Because the twofold interaction was modulated by the factor group follow-up ANOVAs were conducted for each group over both points in time by combining the factors condition and hemisphere.

The follow-up ANOVAs revealed a significant interaction condition^*^hemisphere for the NIMP group, *F*_(2, 32)_ = 7.59, *p* = 0.002, η^2^_*p*_ = 0.32, the main effect condition and the main effect hemisphere were not significant (*p* > 0.05). In the LH NIMP had shorter peak latencies for PW in contrast to W, *t*_(16)_ = −3.35, *p* = 0.004, *d* = 0.81, and PH, *t*_(16)_ = −3.19, *p* = 0.006, *d* = 0.77, peak latencies between W and PH were comparable (*p* > 0.05, see Table 5). No difference between conditions was found in the RH and peak latencies did not differ for none of the conditions between LH and RH (*p* > 0.05). No significant main effect condition, hemisphere and no significant interaction condition^*^hemisphere could be observed for CON and IMP (*p* > 0.05).

### Behavioral results

#### Accuracy

Performance on the PLD—task revealed a main effect group, time and condition, as well as the twofold interactions group^*^condition and time^*^condition (*p* < 0.05, see Table 6, first column).

**Table 6:** **Results of the ANOVAs for repeated measures with *F*-values (df), *p*-values, and effect sizes η^2^_*p*_ for the accuracy and reaction times of the behavioral task including the between-subject factor *group* (CON; IMP; NIMP) and the within-subject-factor *time* (pre; post) and *condition* (W; PH; PW; FF)**.

**Effect**	**Accuracy**			**Reaction times**		
	***F***	***p***	**η^2^_***p***_**	***F***	***p***	**η^2^_***p***_**
Group (G)	31.26 (2, 50)	**<0.001**	0.56	38.06 (2, 50)	**<0.001**	0.60
Time (T)	4.64 (1, 50)	**0.036**	0.09	56.21 (1, 50)	**<0.001**	0.53
Condition (C)	150.76 (2.08, 104.05)	**<0.001**	0.75	382.44 (1.70, 85.10)	**<0.001**	0.88
G*T	0.21 (2, 50)	0.814	–	12.97 (2, 50)	**<0.001**	0.34
G*C	16.89 (4.16, 104.05)	**<0.001**	0.40	37.18 (3.40, 85.10)	**<0.001**	0.60
T*C	6.00 (2.30, 115.21)	**0.002**	0.11	35.05 (2.63, 131.33)	**<0.001**	0.41
G*T*C	1.82 (4.61, 115.21)	0.120	–	6.06 (5.25, 131.33)	**<0.001**	0.20

CON, control children; IMP, improvers; NIMP, non-improvers; pre, before intervention; post, after intervention; W, words; PH, pseudohomophones; PW, pseudowords; FF, false fonts. Significant results are indicated in bold.

**Figure 6:**
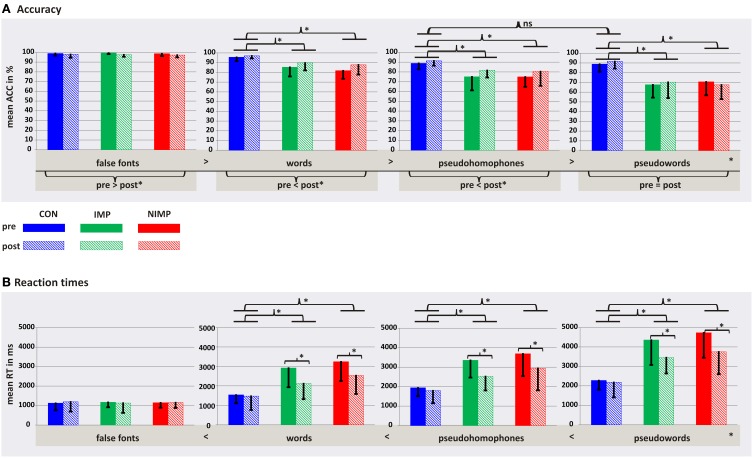
**Behavioral results for the PLD—task for control children (CON), improvers (IMP), and non-improvers (NIMP) before (pre) and after (post) intervention. (A)** Depicts accuracy data and **(B)** illustrates reaction time data. Error bars illustrate standard deviation. ^*^p < 0.05; ns, non-significant.

In order to better understand the two-way interaction between the factors time and condition dependent *post-hoc t*-tests were calculated. Accuracy rates increased over time for W and PH (*p* < 0.05) and slightly decreased for FF (*p* < 0.05). No difference between pre and post was found for PW (*p* > 0.05; see Figure 6A). Furthermore, dependent *post-hoc t*-tests revealed that all children gave more correct answers to FF compared to the linguistic material (W, PH, and PW) before and after intervention (*p* < 0.05). In addition, accuracy rates were pre and post higher for W compared to PH and PW (*p* < 0.05). And all children had higher accuracy rates for PH compared to PW before intervention and after intervention (*p* < 0.05, see Figure 6A).

Dependent *post-hoc t*-tests in order to explain the twofold interaction between group and condition revealed the accuracy pattern FF > W > PH > PW (*p* < 0.05) as described above for IMP and NIMP. In CON, however, no difference between correct answers for PH and PW (*p* > 0.05) could be detected resulting in an accuracy pattern with FF > W > PH = PW (see Figure 6A). Independent *post-hoc t*-tests revealed that over both, pre and post, CON's performance was better to all linguistic stimuli compared to IMP and NIMP (*p* < 0.05). No difference in none of the conditions was found between IMP and NIMP and no group differences were found for FF (*p* > 0.05 see Figure 6A).

#### Reaction times

Performance on the PLD—task revealed a significant main effect group, time and condition, as well as the significant interactions group^*^time, group^*^condition, time^*^condition and group^*^time^*^condition (see Table 6, second column). In order to better understand the threefold interaction separate follow-up ANOVAs were run by combining two of the three factors.

***Follow-up ANOVAs for each point in time***. The analysis before and after intervention revealed a significant main effect group and condition as well as the interaction group^*^condition (*p* < 0.05).

***Follow-up ANOVAs for each condition***. For W, PH, and PW the ANOVAs revealed a significant main effect group and time as well as the interaction group^*^time (*p* < 0.05). No significant effects were found for FF (*p* > 0.05).

***Follow-up ANOVAs for each group***. For CON the analysis revealed a significant main effect condition as well as the interaction condition^*^time (*p* < 0.05) but no main effect time (*p* > 0.05). For IMP and NIMP a significant main effect time and condition and the interaction condition^*^time occurred (*p* < 0.05).

In the following the results of the independent and dependent *post-hoc t*-tests calculated in order to examine the twofold interactions will be summarized.

Independent *post-hoc t*-tests indicated that CON had shorter reactions times to W, PH, and PW compared to IMP and NIMP before intervention and after intervention (*p* < 0.05). No differences for W, PH, and PW were found for the comparison between IMP and NIMP before and after intervention (*p* > 0.05). For FF no group differences were found before and after intervention (*p* > 0.05, see Figure 6B).

Dependent *post-hoc t*-tests within each group revealed the same pattern of reaction times for all groups before and after intervention. CON, IMP, and NIMP had longer reaction times for all linguistic stimuli compared to FF before intervention and after intervention (*p* < 0.05). Furthermore, all groups showed shorter reaction times for W compared to PH and for W compared to PW before and after intervention (*p* < 0.05). And all groups responded slower to PW compared to PH before and after intervention (*p* < 0.05, see Figure 6B).

Reaction times did not change over time in CON for W, PH, PW, and FF (*p* > 0.05). However, IMP and NIMP had faster reaction times after intervention for W, PH, and PW (*p* < 0.05). No changes from pre to post were observed for FF in IMP and NIMP (*p* > 0.05, see Figure 6B).

### Correlational results

When interpreting the correlation results, please note that N300 and N400 mean peak amplitudes have negative values. Larger increase in common word reading fluency was significantly correlated to higher N300 mean peak amplitudes before intervention for W and PH in the RH and PW in the LH and by trend for PW in the RH. Furthermore, a larger increase in pseudoword reading fluency was correlated significantly to higher N300 mean peak amplitudes for W in the RH and by trend for PW in the LH. The linear relationship between N300 before intervention in the RH and gain in common word reading fluency remained stable only in the group of IMP (please see Table 7). Even though only the correlation between N300 mean peak amplitudes before intervention for PH in the RH and increase in common word reading fluency reached significance in the IMP group, the resulting correlations were large, ranging from *r* = −0.54 to *r* = −0.59 (see Table 7). Furthermore, higher N400 mean peak amplitudes after intervention were related to higher N300 mean peak amplitudes before intervention for PW in the LH and by trend for W and PH in the LH in children with DD. In the IMP group higher N400 mean peak amplitudes after intervention were related to higher N300 mean peak amplitudes before intervention for PH and PW in the LH (see Table 7).

**Table 7:** **Pearson correlations across the whole group with DD and within the group of IMP and NIMP between the N300 before intervention and the gain in word and pseudoword reading fluency and the N400 after intervention**.

**Pre**	**Children with DD (*n* = 28)**			**IMP (*n* = 11)**			**NIMP (*n* = 17)**		
	**Post – Pre**		**Post**	**Post – pre**		**Post**	**Post – pre**		**Post**
N300 (μV)	W reading	PW reading	N400 (μV)	W reading	PW reading	N400 (μV)	W reading	PW reading	N400 (μV)
W; LH	−0.13	−0.02	0.34(^*^)	0.32	−0.02	0.33	−0.02	0.13	0.30
PH; LH	−0.32	−0.29	0.35(^*^)	0.19	−0.13	0.85^**^	−0.36	−0.41	0.04
PW; LH	−0.39^*^	−0.34(^*^)	0.38^*^	0.37	−0.40	0.65^*^	−0.02	−0.07	0.15
W; RH	−0.58^**^	−0.41^*^	0.21	−0.54(^*^)	−0.59(^*^)	0.03	0.05	0.02	0.16
PH; RH	−0.46^*^	−0.11	0.15	−0.62^*^	−0.05	0.02	0.15	0.16	0.07
PW; RH	−0.36(^*^)	−0.17	−0.03	−0.41	−0.10	−0.01	−0.02	−0.03	−0.15
Mean; RH	−0.53^**^	−0.26	0.13	−0.58(^*^)	−0.31	0.01	−0.08	−0.06	0.02

DD, developmental dyslexia; IMP, improvers; NIMP, non-improvers; pre, before intervention; post, after intervention; post – pre, difference between pre and post measures; W reading, common word reading fluency from the SLRT II; PW reading, pseudoword reading fluency from the SLRT-II; W, words; PH, pseudohomophones; PW, pseudowords; LH, left hemisphere; RH, right hemisphere;

** *p* < 0.001;

* *p* < 0.05;

(*) p < 0.10.

## Discussion

The aim of the present study was twofold. On the one hand we wanted to clarify whether growth in common word reading fluency during treatment is related to changes in the N400. Furthermore, we were interested whether we could identify pre-existing differences on the neurophysiological level between IMP and NIMP. In order to achieve our aims we investigated a PLD—task before and after children with DD were trained in literacy skills over 6 months. We investigated the ERPs of IMP, who did improve in common word reading fluency for at least half a SD, NIMP who did not show any increase in common word reading fluency and normally developing children.

### Reading improvement is reflected in an increase of N400

As both trainings worked on either orthographic knowledge or GPC we hypothesized to find changes in the N400 (see Introduction), which reflects GPC or the searching process in the orthographic lexicon (Hasko et al., 2013). In line with our previous study (Hasko et al., 2013) we were able to show that both groups of children with DD (IMP and NIMP) had reduced N400 mean peak amplitudes compared to CON before intervention. The reduced N400 amplitudes in IMP and NIMP point to less specified orthographic representations or impairments in accessing the orthographic lexicon or in applying GPC rules (Hasko et al., 2013). As hypothesized a clear trend towards increased N400 amplitudes over time in IMP only was observed. This might indicate an alteration of the process reflected by this component. Thus, in line with previous electrophysiological (Kujala et al., 2001; Santos et al., 2007; Jucla et al., 2009; Penolazzi et al., 2010; Spironelli et al., 2010; Huotilainen et al., 2011; Mayseless, 2011; Lovio et al., 2012) and neuroimaging studies (Simos et al., 2002; Aylward et al., 2003; Temple et al., 2003; Eden et al., 2004; Shaywitz et al., 2004; Simos et al., 2006, 2007b; Richards et al., 2007; Meyler et al., 2008; Richards and Berninger, 2008; Keller and Just, 2009) we found evidence for neurophysiological changes during treatment. This suggests that specific deficient processes in DD, in our case processes related to the N400, are malleable in children with DD. The design of the present study does not allow testing which proportion of reading improvement is related to the applied treatments and which proportion is due to other factors not related to the treatment. Probably due to the small sample size in the IMP group (*n* = 11) the increase in N400 amplitudes, which was moderate to large failed to reach significance. Simulation of the data for a larger sample of IMP revealed a significant increase in the N400 confirming our assumption that the small sample size is the main reason for why the effect does not reach significance.

Due to our classification criterion the common word reading fluency of IMP increased significantly but was still below average after intervention. Therefore, we expected to find increased N400 amplitudes for IMP and thus diminished differences between IMP and CON in N400 amplitudes. However, the differences between IMP and CON were not only diminished after intervention, but absent. N400 amplitudes of CON slightly decreased over time and thus contribute to the absence of differences between IMP and CON, even though this effect does not reach significance. Although no condition effect could be observed, Table 3 shows that the slight decrease in N400 amplitudes is mainly the result of a reduction of the N400 component for W, whereas amplitude means remain stable for PH and PW. A decrease of N400 amplitudes for W in CON is what might be expected with maturation of the reading network. In line with this, it has been found that N400 amplitudes were smaller to orthographic familiar word forms compared to unfamiliar word forms in adults (e.g., Braun et al., 2006; Briesemeister et al., 2009). This suggests that adults in contrast to children (Hasko et al., 2013) adopt different reading strategies for orthographic familiar and unfamiliar word material. In the framework of dual route models of reading (Coltheart et al., 1993, 2001) less effort is needed in order to find a fitting orthographic representation for familiar words in the orthographic lexicon, whereas the search in the orthographic lexicon is prolonged and GPC rules have to be applied in case of unfamiliar word forms resulting in enhanced N400 amplitudes (Hasko et al., 2013). Thus, the observations in the present study might denote the beginning development of the orthographic familiarity effect for the N400 suggesting that some of the W do already possess an entry in the orthographic lexicon and are read via accessing the phonological lexicon directly from the orthographic lexicon in typically developing children. It might be interesting to further investigate when the maturation of the orthographic familiarity effect is fully developed as it indicates the point in time when children steadily use orthographic representations to access phonological representations for familiar word forms.

As expected, children who continued to struggle with common word reading fluency after intervention in our study did not show neurophysiological changes over time. This is consistent with previous research reporting that NIMP continuously display abnormal activation patterns throughout the neuronal reading network (Simos et al., 2007a; Odegard et al., 2008; Davis et al., 2011; Farris et al., 2011; Molfese et al., 2013). One question which remains unanswered is why some children with DD improve during intervention, whereas other do not. This leads directly to our second research question, namely whether there might be any pre-existing differences between IMP and NIMP, which could give insight into improvement and non-improvement.

### Profiling improver and non-improver

Surprisingly, although the hypothesis of neurodiversity within DD has been raised several times (McCandliss and Noble, 2003; Shaywitz et al., 2004; Noble and McCandliss, 2005) neurobiological differences and their influence on improvement in literacy skills during treatment have been neglected in previous intervention studies, thus the analysis run to answer this question in the present study was exploratory. During the inspection of single electrodes and t-maps comparing the topographical distribution between IMP and NIMP we observed a hyperactivation distributed over left and right temporo-frontal electrodes starting around 300 ms after stimulus onset (see Figure 4). Based on the topographical distribution and latency the negative potential was identified as N300. The N300 was investigated employing different tasks and was attributed as being related to grapheme-phoneme conversion (Bentin et al., 1999; Penolazzi et al., 2006), phonological word analysis (Spironelli and Angrilli, 2007, 2009) and the integration of orthographic and phonological representations (Hasko et al., 2012).

In the present study IMP revealed before intervention higher N300 amplitudes for W, PH, and PW in the RH and additionally for PW in the LH compared to NIMP and CON. This suggests that enhanced N300 amplitudes might play an important role for improvement in common word reading fluency, which was further strengthened by our correlational results. Correlations calculated across the whole group of children with DD largely reflected the group differences found for IMP and NIMP, i.e., children who improved in common word reading fluency were those who had higher N300 amplitudes for W, PH, and PW (only marginal significant) in the RH and for PW in the LH before intervention. Especially, higher N300 amplitudes over the RH seem to play an important role for reading improvement as the same pattern of correlation between N300 amplitudes over the RH before intervention and improvement in common word reading fluency was found for IMP only. Children with the highest N300 amplitudes over the RH before intervention displayed also the strongest improvement in common word reading fluency. Even though only the correlation between N300 mean peak amplitudes before intervention for PH in the RH and increase in common word reading fluency reached significance in the IMP group, the resulting correlations were large, ranging from *r* = −0.54 to *r* = −0.59 and are therefore noteworthy.

In previous fMRI studies investigating the PLD—task (Kronbichler et al., 2007; Wimmer et al., 2010) it has been found that this task induces activation throughout the neural reading network including the inferior-frontal subsystem. As mentioned in the introduction evidence for aberrant activation patterns in this subsystem in DD was not as clear as for the left hemispheric posterior subsystem, where hypoactivation was reported repeatedly (Simos et al., 2002; Demonet et al., 2004; Kronbichler et al., 2007; Shaywitz and Shaywitz, 2008; Richlan et al., 2009). With regard to the inferior-frontal subsystem some studies report an hypoactivation (Paulesu et al., 1996; Wimmer et al., 2010; for meta-analyses see: Richlan et al., 2009, 2011); whereas others observed an hyperactivation (Salmelin et al., 1996; Shaywitz et al., 1998; Brunswick et al., 1999; for review see: Pugh et al., 2000; Sandak et al., 2004) in subjects with DD. In line with these inhomogeneous results children with DD in the present study varied with respect to their N300 amplitudes over right and left fronto-temporal electrodes depending on reading improvement or non-improvement with IMP showing significantly higher N300 amplitudes before intervention. It has been suggested that the inferior-frontal subsystem might be involved in articulation processes (Shaywitz and Shaywitz, 2008). Maybe IMP try to adopt different not efficient reading strategies via articulation processes in order to compensate for less specified orthographic representations, impairments in accessing the orthographic lexicon or in applying GPC rules as reflected by reduced N400 amplitudes. This strategy is probably not being applied in the NIMP group, for what reason is unsolved so far.

The observance of pre-existing differences on the neurophysiological level between IMP and NIMP in the present study is in line with the results of Rezaie et al. (2011a,b) who also reported differences between adolescent IMP and NIMP prior to intervention. In contrast to the present study, however, activation profiles of IMP in the studies of Rezaie et al. (2011a,b) seemed to resemble the activation profile of CON. Whereas NIMP were marked by aberrant activation patterns throughout the reading network in contrast to CON, the only difference between IMP and CON was observed in higher activity within the pars opercularis for CON in contrast IMP (Rezaie et al., 2011a,b). This suggests that poor reading skills in NIMP might be stronger influenced by neurobiological factors, whereas for low reading skills in IMP environmental factors like home literacy or socioeconomic status might play an important role. In addition, our results contrast the outcome of Simos et al.'s (2005, 2007a) studies who did not observe differences depending on improvement before intervention. One possible explanation for the absence of neurobiological differences in the study of Simos et al. (2007a) could be the wide age range, as children from 8 to 10 years were included. As this is a very sensitive age for reading development this might probably mask pre-existing differences between IMP and NIMP. Furthermore, in the 2005 study of Simos et al. the NIMP group consisted only of three children allowing to make only descriptive comparisons between IMP and NIMP and thus failing to find pre-existing differences.

Due to the cross-sectional design of the studies of Rezaie et al. (2011a,b), assessing neurobiological activity only before treatment, no statement can be made about neurobiological differences between IMP and NIMP after intervention. And studies comparing IMP and NIMP only after intervention (Odegard et al., 2008; Davis et al., 2011; Farris et al., 2011; Molfese et al., 2013) are limited as it cannot be resolved whether group differences between treatment IMP and NIMP is a cause or the result of improvement. An advantage of the present study is that we have assessed electrophysiological correlates before and after treatment. Interestingly, together with the improvement in reading ability and the increase in the N400 component the N300 amplitudes are higher in IMP compared to CON and NIMP only before intervention. This suggests that the N300 might index a compensatory mechanism or precursor, which facilitates reading improvement as well as the development of the N400 and is given up in favor of the more efficient process reflected by the N400. This is in line with a previous study by Shaywitz et al. (2004) showing that efficient activations throughout the neural reading network were enhanced and compensatory mechanisms were abandoned after a reading intervention. An important role of enhanced N300 amplitudes over the RH for improvement in common word reading fluency as suggested by the correlational results has been hypothesized above. Furthermore, the correlational results indicate that N300 amplitudes over the LH might be related to the increase in the N400. IMP with higher N300 amplitudes over the LH for PH and PW before intervention were those who had higher N400 amplitudes after intervention. Thus, the engagement of the LH seems to be of particular importance for the increase in the N400. At first sight this stands in contrast to our finding that especially the N300 amplitudes over the RH before intervention might be related to reading improvement. In a previous study it has been found that IMP in contrast to NIMP were marked by significantly higher functional connectivity between left and right inferior frontal regions (Farris et al., 2011). The authors suggested that IMP might use the connectivity from LH to RH in order to engage the RH when tasks are difficult. Therefore, with respect to the present study we might hypothesize that enhanced N300 amplitudes over the RH are the result of higher connectivity from LH to RH allowing the engagement of the RH. Thus, it might be concluded that children with highest amplitudes over the LH and highest connectivity between LH and RH show the strongest improvement as indexed by enhanced N400 amplitudes and growth in common word reading fluency. Another explanation might be that the higher LH N300 amplitudes just reflect some additional compensatory mechanism, which is present in IMP only. Because the whole correlational analyses were exploratory no terminal conclusions can be drawn about the relation between the N300 and the increase in common word reading fluency and N400 amplitudes. Future research should further investigate whether the N300 truly has a predictive quality for reading improvement.

When interpreting the above mentioned data it is important to control for group differences on a behavioral level, as these too might influence improvement in literacy skills. Previous studies have reported, that especially, word-reading skills before intervention, phoneme awareness, rapid naming, IQ, and attention have an influence on improvement in literacy skills (Wise et al., 2000; Torgesen et al., 2001). However, in the present study IMP and NIMP had a very similar cognitive profile (see Table 1) suggesting that these factors might play a subordinate role for reading improvement in the present study. Only with respect to reading comprehension IMP differed from NIMP with the latter showing significantly lower reading comprehension skills before and after intervention. Lower performance in reading comprehension might point to deficits in oral language skills. It has been argued that reading comprehension deficits probably arise from poor vocabulary knowledge, weak grammatical skills, and difficulties in oral language comprehension (Snowling and Hulme, 2012a). Furthermore, it has been found that general verbal ability predicts growth in reading ability (Torgesen et al., 2001). Thus, our results suggest that NIMP in addition to deficits in common word reading fluency are marked by stronger impairments in oral language skills in contrast to IMP, impeding reading improvement, and suggesting that NIMP might probably profit from training of oral language skills. Unfortunately, oral language skills were not assessed in this study, therefore this assumption cannot fully be answered.

Previous studies reported that up to 30% of struggling readers do not benefit from intervention (Shanahan and Barr, 1995; Vaughn et al., 2003). With a proportion of 50% our study shows that this number might be even larger. As has been reported above several factors, including word-reading skills before intervention, phoneme awareness, rapid naming, IQ, attention and general verbal ability might influence improvement in literacy skills. Thus, depending on the cognitive profile of children included in the respective studies improvement rates might vary between studies. Furthermore, and most important differences in improvement rates also depend on the operationalization of improvement in literacy skills. Improvement rates will be differing depending on which ability (e.g., phonological awareness, reading fluency, reading comprehension, spelling, etc.) and which cut-off criteria (0.5 SD, 1 SD, median, observation of therapists) is used. So far there are no guidelines or suggested criteria how to define improvement. With respect to the present study we oriented our cut-off criteria on results from current meta-analyses reporting effect sizes of *g* = 0.31 and *g* = 0.33 for reading interventions (Ise et al., 2012; Galuschka et al., 2014).

### Limitations

One limitation of the present study was the quite small sample size of our IMP group, albeit greater (often two times larger) in contrast to many previous studies. Probably due to the small sample size some of the observed effects were only marginally significant. This limits the degree to which the results can be generalized and interpretations have to be drawn cautiously. Therefore, the study needs replications with larger sample sizes. Furthermore, due to small sample sizes, splitting our groups according to type of intervention (IP1 vs. IP2) was not reasonable. Therefore, the present study does not allow discriminating intervention effects depending on the type of treatment. Future studies investigating treatment IMP and NIMP need to take into account that groups will be divided in two and that depending on the definition of improvement in literacy skills some children might be excluded from the study, meaning very large sample sizes are needed.

## Conclusion

In the present study we attempted to investigate the ERPs related to reading improvement. To summarize, children who significantly improve in reading during intervention are marked by an increased N400 component, which reflects GPC or the searching process within the orthographic lexicon. Children who continue to struggle in reading do not exhibit any neurophysiological changes over time. Furthermore, IMP and NIMP can be discriminated according to their neurophysiological profile already before intervention. Only IMP display higher N300 mean peak amplitudes over right fronto-temporal electrodes when processing W, PH, and PW and additionally over left fronto-temporal electrodes for PW. The importance of N300 amplitudes for reading improvement is strengthened by the correlational results in the IMP group. The higher the N300 amplitudes over the RH before intervention the larger the improvement in common word reading fluency. Furthermore, IMP with higher N300 amplitudes over the LH before intervention have higher N400 amplitudes after intervention. After intervention the N300 of IMP is equally high to the N300 of CON and NIMP suggesting that the N300 might index a compensatory mechanism or precursor, which facilitates the development of the N400 as well as reading improvement.

Future research should concentrate on the examination of the special needs of NIMP. What are the factors that make them more resistant to environmental change? Do they exhibit a different type of DD and therefore have to be treated in a different way? But how can this be identified? Which role play genetic differences for reading improvement? With respect to the present study NIMP seem to be a special group, who might benefit from another type of training. Lower reading comprehension skills in NIMP in the present study point to more pronounced impairments in oral language skills in contrast to IMP. Therefore, the NIMP in the present study might possibly profit from an additional training in oral language skills (Snowling and Hulme, 2011, 2012b). Answering these questions would help enormously to improve and adjust intervention for children with DD.

Important for all future studies, is to keep in mind that children with DD, even though matched with respect to their cognitive profile might differ regarding their neuronal profile. In fact, it is extremely difficult to categorize children on the behavioral level when the underlying cause of their DD might be very different with contributions from neurophysiology, neurobiology, genetics and environment. Future intervention studies should carefully distinguish between IMP and NIMP as the mixture of these children might even distort the results.

One of the main future goals is to further examine the N300 effects and to verify whether they can be replicated and hold true for a large sample size. Furthermore, future research should investigate whether the N300 might be a predictor for reading improvement in response to treatment. If the N300 truly has a predictive quality for response to intervention then it would be possible to streamline therapies for certain children.

### Conflict of interest statement

The authors declare that the research was conducted in the absence of any commercial or financial relationships that could be construed as a potential conflict of interest.

## References

[B1] Achenbach T. M. (1991). Manual for the Child Behavior Checklist/4–18 and 1991 Profile. Burlington: University of Vermont, Department of Psychiatry

[B2] APA (2013). Diagnostic and Statistical Manual of Mental Disorders, 5th Edn., DSM-5. Washington, DC: American Psychiatric Association

[B3] Arnold E. M. Goldston D. B. Walsh A. K. Reboussin B. A. Daniel S. S. Hickman E. (2005). Severity of emotional and behavioral problems among poor and typical readers. J. Abnorm. Child Psychol. 33, 205–217 10.1007/s10802-005-1828-9 15839498

[B4] Aylward E. H. Richards T. L. Berninger V. W. Nagy W. E. Field K. M. Grimme A. C. (2003). Instructional treatment associated with changes in brain activation in children with dyslexia. Neurology 61, 212–219 10.1212/01.WNL.0000068363.05974.64 12874401

[B5] Bentin S. Mouchetant-Rostaing Y. Giard M. H. Echallier J. F. Pernier J. (1999). ERP manifestations of processing printed words at different psycholinguistic levels: time course and scalp distribution. J. Cogn. Neurosci. 11, 235–260 10.1162/089892999563373 10402254

[B6] Birkel P. (1994). Weingartener Grundwortschatz Rechtschreib-Test für Zweite und Dritte Klassen (WRT2+). Göttingen: Hogrefe

[B7] Birkel P. (2007). Weingartener Grundwortschatz Rechtschreib-Test für Dritte und Vierte Klassen (WRT3+). Göttingen: Hogrefe

[B8] Braun M. Jacobs A. M. Hahne A. Ricker B. Hofmann M. Hutzler F. (2006). Model-generated lexical activity predicts graded ERP amplitudes in lexical decision. Brain Res. 1073–1074, 431–439 10.1016/j.brainres.2005.12.078 16464440

[B9] Briesemeister B. B. Hofmann M. J. Tamm S. Kuchinke L. Braun M. Jacobs A. M. (2009). The pseudohomophone effect: evidence for an orthography-phonology-conflict. Neurosci. Lett. 455, 124–128 10.1016/j.neulet.2009.03.010 19368860

[B10] Brunswick N. McCrory E. Price C. J. Frith C. D. Frith U. (1999). Explicitly and implicit processing of words and pseudowords by adult developmental dyslexics. Brain 122, 1901–1917 10.1093/brain/122.10.1901 10506092

[B11] Bühner M. Ziegler M. (2009). Statistik für Psychologen und Sozialwissenschaftler. München: Pearson

[B12] Cattell R. B. Weiß R. H. Osterland J. (1997). Grundintelligenztest Skala 1 (CFT 1). Göttingen: Hogrefe

[B13] Caylak E. (2009). Neurobiological approaches on brains of children with dyslexia: review. Acad. Radiol. 16, 1003–1024 10.1016/j.acra.2009.02.012 19406674

[B14] Cohen J. (1988). Statistical Power Analysis for the Behavioural Sciences. Hillsdale, NJ: Lawrence Erlbaum

[B15] Coltheart M. Curtis B. Atkins P. Haller M. (1993). Models of reading aloud: dual-route and parallel-distributed-processing approaches. Psychol. Rev. 100, 589–608 10.1037/0033-295X.100.4.589

[B16] Coltheart M. Rastle K. Perry C. Langdon R. Ziegler J. (2001). DRC: a dual route cascaded model of visual word recognition and reading aloud. Psychol. Rev. 108, 204–256 10.1037/0033-295X.108.1.204 11212628

[B17] Daniel S. S. Walsh A. K. Goldston D. B. Arnold E. M. Reboussin B. A. Wood F. B. (2006). Suicidality, school dropout and reading problems among adolescents. J. Learn. Disabil. 39, 507–514 10.1177/00222194060390060301 17165618

[B18] Davis N. Barquero L. Compton D. L. Fuchs L. S. Fuchs D. Gore J. C. (2011). Functional correlates of children's responsiveness to intervention. Dev. Neuropsychol. 36, 288–301 10.1080/87565641.2010.549875 21462008 PMC4416061

[B19] Deacon D. Dynowska A. Ritter W. Grose-Fifer J. (2004). Repetition and semantic priming of nonwords: implications for the theories of N400 and word recognition. Psychophysiology 41, 60–74 10.1111/1469-8986.00120 14693001

[B20] Demonet J. F. Taylor M. J. Chaix Y. (2004). Developmental dyslexia. Lancet 363, 1451–1460 10.1016/S0140-6736(04)16106-0 15121410

[B21] Dummer-Smoch L. Hackethal R. (2007). Kieler Leseaufbau. Kiel: Veris

[B22] Eden G. F. Jones K. M. Cappell K. Gareau L. Wood F. B. Zeffiro T. A. (2004). Neural changes following remediation in adult developmental dyslexia. Neuron 44, 411–422 10.1016/j.neuron.2004.10.019 15504323

[B26] Electrical Geodesics Inc (2007). Geodesic Sensor Net Technical Manual. Available online at: http://ganesha.uoregon.edu/images/8/8c/Gsn013107.pdf?bcsiscan64377d2312a1e457=0andbcsiscanfilename=Gsn013107.pdf (Accessed January 24, 2014).

[B23] Esser G. Wyschkon A. Schmidt M. H. (2002). Was wird aus Achtjährigen mit einer Lese-Rechtschreibstörung. Z. Klin. Psychol. Psychother. 31, 235–242 10.1026/1616-3443.31.4.235

[B24] Farris E. A. Odegard T. N. Miller H. L. Ring J. Allen G. Black J. (2011). Functional connectivity between the left and right inferior frontal lobes in a small sample of children with and without reading difficulties. Neurocase 17, 425–439 10.1080/13554794.2010.532141 21590585

[B25] Galuschka K. Ise E. Krick K. Schulte-Körne G. (2014). Effectiveness of treatment approaches for children and adolescents with reading disabilities: a meta-analysis of randomized controlled trials. PLoS ONE 9:e89900 10.1371/journal.pone.0089900 24587110 PMC3935956

[B27] Goldston D. B. Walsh A. Mayfield Arnold E. Reboussin B. Sergent Daniel S. Erkanli A. (2007). Reading problems, psychiatric disorders, and functional impairment from mid- to late adolescence. J. Am. Acad. Child Adolesc. Psychiatry 46, 25–32 10.1097/01.chi.0000242241.77302.f4 17195726

[B28] Groth K. Hasko S. Bruder J. Kunze S. Schulte-Körne G. (2013). Interventionseffekte bei Lese-Rechtschreibstörung: evaluation von zwei Förderkonzepten unter besonderer Betrachtung methodischer Aspekte. Lernen und Lernstörungen 2, 161–175 10.1024/2235-0977/a000038

[B29] Hasko S. Bruder J. Bartling J. Schulte-Körne G. (2012). N300 indexes deficient integration of orthographic and phonological representations in children with dyslexia. Neuropsychologia 50, 640–654 10.1016/j.neuropsychologia.2012.01.001 22245008

[B30] Hasko S. Groth K. Bruder J. Bartling J. Schulte-Körne G. (2013). The time course of reading processes in children with and without dyslexia: an ERP study. Front. Hum. Neurosci. 7:570 10.3389/fnhum.2013.00570 24109444 PMC3791381

[B31] Hoffmann S. Falkenstein M. (2008). The correction of eye blink artefacts in the EEG: a comparison of two prominent methods. PLoS ONE 3:e3004 10.1371/journal.pone.0003004 18714341 PMC2500159

[B32] Huotilainen M. Lovio R. Kujala T. Tommiska V. Karma K. Fellman V. (2011). Could audiovisual training be used to improve cognition in extremely low birth weight children? Acta Paediatr. 100, 1489–1494 10.1111/j.1651-2227.2011.02345.x 21535135

[B33] Ise E. Engel R. R. Schulte-Körne G. (2012). Was hilft bei Lese-Rechtschreibstörung? Ergebnisse einer Metaanalyse zur Wirksamkeit deutschsprachiger Förderansätze. Kindheit und Entwicklung 21, 122–136 10.1026/0942-5403/a000077

[B34] Ise E. Schulte-Körne G. (2010). Spelling deficits in dyslexia: evaluation of an orthographic spelling training. Ann. Dyslexia 60, 18–39 10.1007/s11881-010-0035-8 20352378

[B35] Jucla M. Nenert R. Chaix Y. Demonet J. F. (2009). Remediation effects on N170 and P300 in children with developmental dyslexia. Behav. Neurol. 22, 121–129 10.1155/2010/913692 20595744 PMC5434325

[B36] Katusic S. K. Colligan R. C. Barbaresi W. J. Schaid D. J. Jacobsen S. J. (2001). Incidence of reading disability in a population-based birth cohort, 1976-1982, Rochester, Minn. Mayo Clin. Proc. 76, 1081–1092 10.4065/76.11.1081 11702896

[B37] Keller T. A. Just M. A. (2009). Altering cortical connectivity: remediation-induced changes in the white matter of poor readers. Neuron 64, 624–631 10.1016/j.neuron.2009.10.018 20005820 PMC2796260

[B38] Kronbichler M. Bergmann J. Hutzler F. Staffen W. Mair A. Ladurner G. (2007). Taxi vs. taksi: on orthographic word recognition in the left ventral occipitotemporal cortex. J. Cogn. Neurosci. 19, 1584–1594 10.1162/jocn.2007.19.10.1584 17933023 PMC2989180

[B39] Kujala T. Karma K. Ceponiene R. Belitz S. Turkkila P. Tervaniemi M. (2001). Plastic neural changes and reading improvement caused by audiovisual training in reading-impaired children. Proc. Natl. Acad. Sci. U.S.A. 98, 10509–10514 10.1073/pnas.181589198 11517333 PMC56991

[B40] Kutas M. Federmeier K. D. (2011). Thirty years and counting: finding menaing in the N400 component of the event-related brain potential (ERP). Annu. Rev. Psychol. 62, 621–641 10.1146/annurev.psych.093008.131123 20809790 PMC4052444

[B41] Lau E. F. Philips C. Poeppel D. (2008). A cortical network for semantics: (De)constructiong the N400. Nat. Rev. Neurosci. 9, 920–933 10.1038/nrn2532 19020511

[B42] Lenhard W. Schneider W. (2006). Ein Leseverständnistest für Erst- bis Sechstklässler (ELFE1-6). Göttingen: Hogrefe

[B43] Lovio R. Halttunen A. Lyytinen H. Näätänen R. Kujala T. (2012). Reading skill and neural processing accuracy improvement after a 3-hour intervention in preschoolers with difficulties in reading-related skills. Brain Res. 1448, 42–55 10.1016/j.brainres.2012.01.071 22364735

[B44] Luck S. J. (2005). An Introduction to Event-Related Potential Technique. Cambridge, London: The MIT Press

[B45] Mayseless N. (2011). Can intervention programs influence how the dyslexic brain processes low-level visual stimuli? Dev. Neuropsychol. 36, 949–954 10.1080/87565641.2011.606421 21978016

[B46] McArthur G. Eve P. M. Jones K. Banales E. Kohnen S. Anandakumar T. (2012). Phonics training for English-speaking poor readers. Cochrane Database Syst. Rev. 12:CD009115 10.1002/14651858.CD009115.pub2 23235670

[B47] McCandliss B.-D. Noble K.-G. (2003). The development of reading impairment: a cognitive neuroscience model. Ment. Retard. Dev. Disabil. Res. Rev. 9, 196–204 10.1002/mrdd.10080 12953299

[B48] Meyler A. Keller T. A. Cherkassky V. L. Gabrieli J. D. Just M. A. (2008). Modifying the brain activation of poor readers during sentence comprehension with extended remedial instruction: a longitudinal study of neuroplasticity. Neuropsychologia 46, 2580–2592 10.1016/j.neuropsychologia.2008.03.012 18495180 PMC2598765

[B49] Molfese P. J. Fletcher J. M. Denton C. A. (2013). Adequate versus inadequate response to reading intervention: an event-related potentials assessment. Dev. Neuropsychol. 38, 534–549 10.1080/87565641.2013.825260 24219694 PMC3891574

[B50] Moll K. Landerl K. (2010). SLRT-II. Lese- und Rechtschreibtest. Weiterentwicklung des Salzburger Lese- und Rechtschreibtests (SLRT). Bern: Hans Huber

[B51] Mugnaini D. Lassi S. La Malfa G. Albertini G. (2009). Internalizing correlates of dyslexia. World J. Pediatr. 5, 255–264 10.1007/s12519-009-0049-7 19911139

[B52] National Institute of Child Health and Human Development (2000). Report of the National Reading Panel. Teaching Children to Read: an Evidence-Based Assessment of the Scientific Research Literature on Reading and its Implications for Reading Instruction: Reports of the Subgroups (NIH Publication No. 00-4754). Washington, DC: Government Printing Office

[B53] Noble K. G. McCandliss B. D. (2005). Reading development and impairment: behavioral, social, and neurobiological factors. J. Dev. Behav. Pediatr. 26, 370–378 10.1097/00004703-200510000-00006 16222178

[B54] Odegard T. N. Ring J. Smith S. Biggan J. Black J. (2008). Differentiating the neural response to intervention in children with developmental dyslexia. Ann. Dyslexia 58, 1–14 10.1007/s11881-008-0014-5 18483867

[B55] Paulesu E. Frith U. Snowling M. Gallagher A. Morton J. Frackowiak R. S. J. (1996). Is developmental dyslexia a disconnection syndrome?: evidence from PET scanning. Brain 119, 143–157 10.1093/brain/119.1.143 8624677

[B56] Penolazzi B. Spironelli C. Vio C. Angrilli A. (2006). Altered hemispheric asymmetry during word processing in dyslexic children: an event-related potential study. Neuroreport 17, 429–433 10.1097/01.wnr.0000203350.99256.7d 16514371

[B57] Penolazzi B. Spironelli C. Vio C. Angrilli A. (2010). Brain plasticity in developmental dyslexia after phonological treatment: a beta EEG band study. Behav. Brain Res. 209, 179–182 10.1016/j.bbr.2010.01.029 20109496

[B58] Petermann F. Petermann U. (2007). Hamburg-Wechsler-Intelligenztest für Kinder- IV - HAWIK-IV. Bern: Hans Huber

[B59] Pugh K. R. Mencl W. E. Jenner A. R. Katz L. Frost S. J. Lee J. R. (2000). Functional neuroimaging studies of reading and reading disability (developmental dyslexia). Ment. Retard. Dev. Disabil. Res. Rev. 6, 207–213 10.1002/1098-2779(2000)6:3<207::AID-MRDD8>3.0.CO;2-P 10982498

[B60] Rezaie R. Simos P. G. Fletcher J. M. Cirino P. T. Vaughn S. Papanicolaou A. C. (2011a). Engagement of temporal lobe regions predicts response to educational interventions in adolescent struggling readers. Dev. Neuropsychol. 36, 869–888 10.1080/87565641.2011.606404 21978010 PMC3308683

[B61] Rezaie R. Simos P. G. Fletcher J. M. Cirino P. T. Vaughn S. Papanicolaou A. C. (2011b). Temporo-parietal brain activity as a longitudinal predictor of response to educational interventions among middle school struggling readers. J. Int. Neuropsychol. Soc. 17, 875–885 10.1017/S1355617711000890 21740612 PMC3174865

[B62] Richards T. Berninger V. Winn W. Stock P. Wagner R. Muse A. (2007). Functional MRI activation in children with and without dyslexia during pseudoword aural repeat and visual decode: before and after treatment. Neuropsychology 21, 732–741 10.1037/0894-4105.21.6.732 17983287

[B63] Richards T. L. Berninger V. W. (2008). Abnormal fMRI connectivity in children with dyslexia during a phoneme task: before but not after treatment. J. Neurolinguist. 21, 294–304 10.1016/j.jneuroling.2007.07.002 19079567 PMC2597820

[B64] Richlan F. (2012). Developmental dyslexia: dysfunction of a left hemisphere reading network. Front. Hum. Neurosci. 6:120 10.3389/fnhum.2012.00120 22557962 PMC3340948

[B65] Richlan F. Kronbichler M. Wimmer H. (2009). Functional abnormalities in the dyslexic brain: a quantitative meta-analysis of neuroimaging studies. Hum. Brain Mapp. 30, 3299–3308 10.1002/hbm.20752 19288465 PMC2989182

[B66] Richlan F. Kronbichler M. Wimmer H. (2011). Meta-analyzing brain dysfunctions in dyslexic children and adults. Neuroimage 56, 1735–1742 10.1016/j.neuroimage.2011.02.040 21338695

[B67] Richlan F. Kronbichler M. Wimmer H. (2013). Structural abnormalities in the dyslexic brain: a meta-analysis of voxel-based morphometry studies. Hum. Brain Mapp. 34, 3055–3065 10.1002/hbm.22127 22711189 PMC6870307

[B68] Salmelin R. Kiesilä P. Uutela K. Service E. Salonen O. (1996). Impaired visual word processing in dyslexia revealed with magnetoencephalography. Ann. Neurol. 40, 157–162 10.1002/ana.410400206 8773596

[B69] Sandak R. Mencl W. E. Frost S. J. Pugh K. R. (2004). The neurobiological basis of skilled and impaired reading: recent findings and new directions. Sci. Stud. Read. 8, 273–292 10.1207/s1532799xssr0803_6

[B70] Santos A. Joly-Pottuz B. Moreno S. Habib M. Besson M. (2007). Behavioural and event-related potentials evidence for pitch discrimination deficits in dyslexic children: improvement after intensive phonic intervention. Neuropsychologia 45, 1080–1090 10.1016/j.neuropsychologia.2006.09.010 17140611

[B71] Schulte-Körne G. Bruder J. Ise E. Rückert E. (2012). Spelling disability - neurophysiologic correlates and intervention, in Reading, Writing, Mathematics and the Developing Brain: Listening to Many Voices. Literacy Studies, eds Breznitz Z. Rubinsten O. Molfese V. J. Molfese D. L. (Dordrecht; Heidelberg; New York; London: Springer), 157–175 10.1007/978-94-007-4086-0_9

[B72] Schulte-Körne G. Deimel W. Remschmidt H. (2001). Zur Diagnostik der Lese-Rechtschreibstörung. Z. Kinder Jugendpsychiatr. Psychother. 29, 113–116 10.1024/1422-4917.29.2.113 11393049

[B73] Schulte-Körne G. Mathwig F. (2007). Das Marburger Rechtschreibtraining. Ein Regelgeleitetes Förderprogramm für Rechtschreibschwache Kinder. Bochum: Winkler

[B74] Schurz M. Sturm D. Richlan F. Kronbichler M. Ladurner G. Wimmer H. (2010). A dual-route perspective on brain activation in response to visual words: evidence for a length by lexicality interaction in the visual word form area (VWFA). Neuroimage 49, 2649–2661 10.1016/j.neuroimage.2009.10.082 19896538 PMC2989181

[B75] Shanahan T. Barr R. (1995). Reading Recovery: an independent evaluation of the effects of an early instructional intervention for at-risk learners. Read. Res. Q. 30, 968–996 10.2307/748206

[B76] Shaywitz B. A. Shaywitz S. E. Blachman B. A. Pugh K. R. Fulbright R. K. Skudlarski P. (2004). Development of left occipitotemporal systems for skilled reading in children after a phonologically- based intervention. Biol. Psychiatry 55, 926–933 10.1016/j.biopsych.2003.12.019 15110736

[B77] Shaywitz B. A. Shaywitz S. E. Pugh K. R. Mencl W. E. Fulbright R. K. Skudlarski P. (2002). Disruption of posterior brain systems for reading in children with developmental dyslexia. Biol. Psychiatry 52, 101–110 10.1016/S0006-3223(02)01365-3 12114001

[B78] Shaywitz S. E. Fletcher J. M. Holahan J. M. Schneider A. Marchione K. E. Stuebing K. K. (1999). Persistence of dyslexia: the connecticut longitudinal study at adolescence. Pediatrics 104, 1351–1359 10.1542/peds.104.6.1351 10585988

[B79] Shaywitz S. E. Gruen J. R. Shaywitz B. A. (2007). Management of dyslexia, its rationale, and underlying neurobiology. Pediatr. Clin. North Am. 54, 609–623 10.1016/j.pcl.2007.02.013 17543912

[B80] Shaywitz S. E. Shaywitz B. A. (2008). Paying attention to reading: the neurobiology of reading and dyslexia. Dev. Psychopathol. 20, 1329–1349 10.1017/S0954579408000631 18838044

[B81] Shaywitz S. E. Shaywitz B. A. Fletcher J. M. Escobar M. D. (1990). Prevalence of reading disability in boys and girls results of the Connecticut longitudinal study. J. Am. Med. Assoc. 264, 998–1002 10.1001/jama.1990.03450080084036 2376893

[B82] Shaywitz S. E. Shaywitz B. A. Pugh K. R. Fulbright R. K. Constable R. T. Mencl W. E. (1998). Functional disruption in the organization of the brain for reading in dyslexia. Proc. Natl. Acad. Sci. U.S.A. 95, 2636–2641 10.1073/pnas.95.5.2636 9482939 PMC19444

[B83] Simos P. G. Fletcher J. M. Bergman E. Breier J. I. Foorman B. R. Castillo E. M. (2002). Dyslexia-specific brain activation profile becomes normal following successful remedial training. Neurology 58, 1203–1213 10.1212/WNL.58.8.1203 11971088

[B84] Simos P. G. Fletcher J. M. Denton C. Sarkari S. Billingsley-Marshall R. Papanicolaou A. C. (2006). Magnetic source imaging studies of dyslexia interventions. Dev. Neuropsychol., 30, 591–611 10.1207/s15326942dn3001_4 16925476

[B85] Simos P. G. Fletcher J. M. Sarkari S. Billingsley R. L. Denton C. Papanicolaou A. C. (2007a). Altering the brain circuits for reading through intervention: a magnetic source imaging study. Neuropsychology 21, 485–496 10.1037/0894-4105.21.4.485 17605581

[B86] Simos P. G. Fletcher J. M. Sarkari S. Billingsley R. L. Francis D. J. Castillo E. M. (2005). Early development of neurophysiological processes involved in normal reading and reading disability: a magnetic source imaging study. Neuropsychology 19, 787–798 10.1037/0894-4105.19.6.787 16351354

[B87] Simos P. G. Fletcher J. M. Sarkari S. Billingsley-Marshall R. Denton C. A. Papanicolaou A. C. (2007b). Intensive instruction affects brain magnetic activity associated with oral word reading in children with persistent reading disabilities. J. Learn. Disabil. 40, 37–48 10.1177/00222194070400010301 17274546

[B88] Snowling M. J. Hulme C. (2011). Evidence-based interventions for reading and language difficulties: creating a virtuous circle. Br. J. Educ. Psychol. 81, 1–23 10.1111/j.2044-8279.2010.02014.x 21391960

[B89] Snowling M. J. Hulme C. (2012a). Annual research review: the nature and classification of reading disorders-a commentary on proposals for DSM-5. J. Child Psychol. Psychiatry 53, 593–607 10.1111/j.1469-7610.2011.02495.x 22141434 PMC3492851

[B90] Snowling M. J. Hulme C. (2012b). Interventions for children's language and literacy difficulties. Int. J. Lang. Commun. Disord. 47, 27–34 10.1111/j.1460-6984.2011.00081.x 22268899 PMC3429860

[B91] Spironelli C. Angrilli A. (2007). Influence of phonological, semantic and orthographic tasks on the early linguistic components N150 and N350. Int. J. Psychophysiol. 64, 190–198 10.1016/j.ijpsycho.2007.02.002 17363097

[B92] Spironelli C. Angrilli A. (2009). Developmental aspects of automatic word processing: language lateralization of early ERP components in children, young adults and middle-aged subjects. Biol. Psychol. 80, 35–45 10.1016/j.biopsycho.2008.01.012 18343558

[B93] Spironelli C. Penolazzi B. Vio C. Angrilli A. (2010). Cortical reorganization in dyslexic children after phonological training: evidence from early evoked potentials. Brain 133, 3385–3395 10.1093/brain/awq199 20688811

[B94] Temple E. Deutsch G.-K. Poldrack R.-A. Miller S. Tallal P. Merzenich M.-M. (2003). Neural deficits in children with dyslexia ameliorated by behavioral remediation: evidence from functional MRI. Proc. Natl. Acad. Sci. U.S.A. 100, 2860–2865 10.1073/pnas.0030098100 12604786 PMC151431

[B95] Torgesen J. K. Alexander A. W. Wagner R. K. Rashotte C. A. Voeller K. K. S. Conway T. (2001). Intensive remedial instruction for children with severe reading disabilities: immediate and long-term outcomes from two instructional approaches. J. Learn. Disabil. 34, 35–58 10.1177/002221940103400104 15497271

[B96] van der Mark S. Bucher K. Maurer U. Schulz E. Brem S. Buckelmüller J. (2009). Children with dyslexia lack multiple specializations along the visual word-form (VWF) system. Neuroimage 47, 1940–1949 10.1016/j.neuroimage.2009.05.021 19446640

[B97] van der Mark S. Klaver P. Bucher K. Maurer U. Schulz E. Brem S. (2011). The left occipitotemporal system in reading: disruption of focal fMRI connectivity to left inferior frontal and inferior parietal language areas in children with dyslexia. Neuroimage 54, 2426–2436 10.1016/j.neuroimage.2010.10.002 20934519

[B98] Vaughn S. Linan-Thompson S. Hickman P. (2003). Response to instruction as a means of identifying students with reading/learning disabilities. Except. Children 69, 391–409

[B99] Weiss S. Grabner R. H. Kargl R. Purgstaller C. Fink A. (2010). Behavioral and neurophysiological effects of morphological awareness training on spelling and reading. Read. Writ. 23, 645–671 10.1007/s11145-009-9177-7

[B100] Willcutt E. Pennington B. (2000). Psychiatric comorbidity in children and adolescents with reading disability. J. Child Psychol. Psychiatry 41, 1039–1048 10.1111/1469-7610.00691 11099120

[B101] Willcutt E. G. Betjemann R. S. Pennington B. F. Olson R. K. Defries J. C. Wadsworth S. J. (2007). Longitudinal study of reading disability and attention-deficit/ hyperactivity disorder: implications for education. Mind Brain Educ. 1, 181–192 10.1111/j.1751-228X.2007.00019.x

[B102] Wimmer H. Schurz M. Sturm D. Richlan F. Klackl J. Kronbichler M. (2010). A dual-route perspective on poor reading in a regular orthography: an fMRI study. Cortex 46, 1284–1298 10.1016/j.cortex.2010.06.004 20650450 PMC3073233

[B103] Wise B. W. Ring J. Olson R. K. (2000). Individual differences in gains from computer-assisted remedial reading. J. Exp. Child Psychol. 77, 197–235 10.1006/jecp.1999.2559 11023657

[B104] Zhou W. Zhou J. Zhao H. Ju L. (2005). Removing eye movement and power line artifacts from the EEG based on ICA. Conf. Proc. IEEE Eng. Med. Biol. Soc. 6, 6017–6020 10.1109/IEMBS.2005.1615863 17281633

